# Chemical and biosynthetic potential of *Penicillium shentong* XL-F41

**DOI:** 10.3762/bjoc.20.52

**Published:** 2024-03-15

**Authors:** Ran Zou, Xin Li, Xiaochen Chen, Yue-Wei Guo, Baofu Xu

**Affiliations:** 1 Shandong Laboratory of Yantai Drug Discovery, Bohai Rim Advanced Research Institute for Drug Discovery, Yantai, Shandong 264117, China; 2 School of Life Sciences, Ludong University, Yantai 264025, China; 3 State Key Laboratory of Drug Research, Shanghai Institute of Materia Medica, Chinese Academy of Sciences, 555 Zu Chong Zhi Road, Zhangjiang Hi-Tech Park, Shanghai, 201203, China

**Keywords:** genome analysis, indole terpene alkaloid, natural products, *Penicillium*, structure elucidation

## Abstract

*Penicillium* strains are renowned for producing diverse secondary metabolites with unique structures and promising bioactivities. Our chemical investigations, accompanied by fermentation media optimization, of a newly isolated fungus, *Penicillium shentong* XL-F41, led to the isolation of twelve compounds. Among these are two novel indole terpene alkaloids, shentonins A and B (**1** and **2**), and a new fatty acid **3**. Shentonin A (**1**) is distinguished by an unusual methyl modification at the oxygen atom of the typical succinimide ring, a feature not seen in the structurally similar brocaeloid D. Additionally, shentonin A (**1**) exhibits a *cis* relationship between H-3 and H-4, as opposed to the *trans* configuration in brocaeloid D, suggesting a divergent enzymatic ring-expansion process in their respective fungi. Both shentonins A (**1**) and B (**2**) also feature a reduction of a carbonyl to a hydroxy group within the succinimide ring. All isolated compounds were subjected to antimicrobial evaluations, and compound **12** was found to have moderate inhibitory activity against *Candia albicans*. Moreover, genome sequencing of *Penicillium shentong* XL-F41 uncovered abundant silent biosynthetic gene clusters, indicating the need for future efforts to activate these clusters and unlock the full chemical potential of the fungus.

## Introduction

*Penicillium*, a genus within the *Ascomycota* phylum, is a type of critical saprophytic fungus with over 400 strains identified in diverse environments such as mountains, oceans, and the human gut [1]. After the first antibiotic mycophenolic acid originally isolated by Gosio in the 1890s [2], the important antibiotic penicillin was characterized more than one decade after Fleming discovered the antibacterial activity of a *Penicillium* extract, and since then, *Penicillium* has been an important target in drug development. Researchers have identified numerous compounds with anticancer properties, including mycophenolic acid, brefeldin A, and wortmannin [3], as well as compounds with antibacterial properties like xestodecalactones A–C, penicifurans A, and anthraquinone-citrinin [4]. From 2010 to 2022, researchers have identified over 260 secondary metabolites from *Penicillium* [5], exhibiting not only antibacterial and anticancer activities but also potent antioxidant properties, inhibition of GSK-3β and α-glucosidase activities, and interaction with the pregnane X receptor (PXR). These compounds are categorized into polyketides, alkaloids, sterol derivatives, terpenoids, and macrolides, with polyketides and alkaloids comprising 40% and 32% of the total, respectively.

Alkaloids are a diverse group of compounds with multiple pharmacological activities, including anti-inflammatory, antibacterial, antiviral, insecticidal, and anticancer properties [6–7]. Historically, most alkaloids were isolated from higher plants, with a significant number found in the *Apocynaceae* family. Notable examples such as vinblastine, vinorelbine, vincristine, and vindesine have gained prominence as effective anticancer drugs [6–10]. Recent studies have revealed that certain fungi are also prolific sources of indole alkaloids, which are among the largest classes of nitrogen-containing secondary metabolites. Characterized by at least one indole moiety and derived from tryptophan or tryptamine, indole alkaloids are known for their diverse structures, electron-donating capabilities, and excellent biocompatibility, contributing to their potent antibacterial and anticancer activities [9–10]. Over 4000 species [8] producing indole alkaloids have been identified, and many of these compounds are now successfully employed in clinical applications.

Despite the extensive catalog of secondary metabolites discovered, the pace of new findings has decelerated. However, the advent of bioinformatics analysis tools has reinvigorated the search for fungal secondary metabolites. The estimated number of non-redundant clusters in *Penicillium* is around 25,000 [1], yet the number of isolated compounds is significantly lower, indicating the presence of many unexpressed gene clusters. This suggests a wealth of undiscovered compounds with potentially novel structures and significant biological activities. To stimulate the expression of biosynthetic gene clusters (BGCs), several methods can be utilized, for instance, epigenetic regulation, co-culture, precursor feeding, heterologous expression, and changing fermentation parameters [11–14].

**Figure 1:**
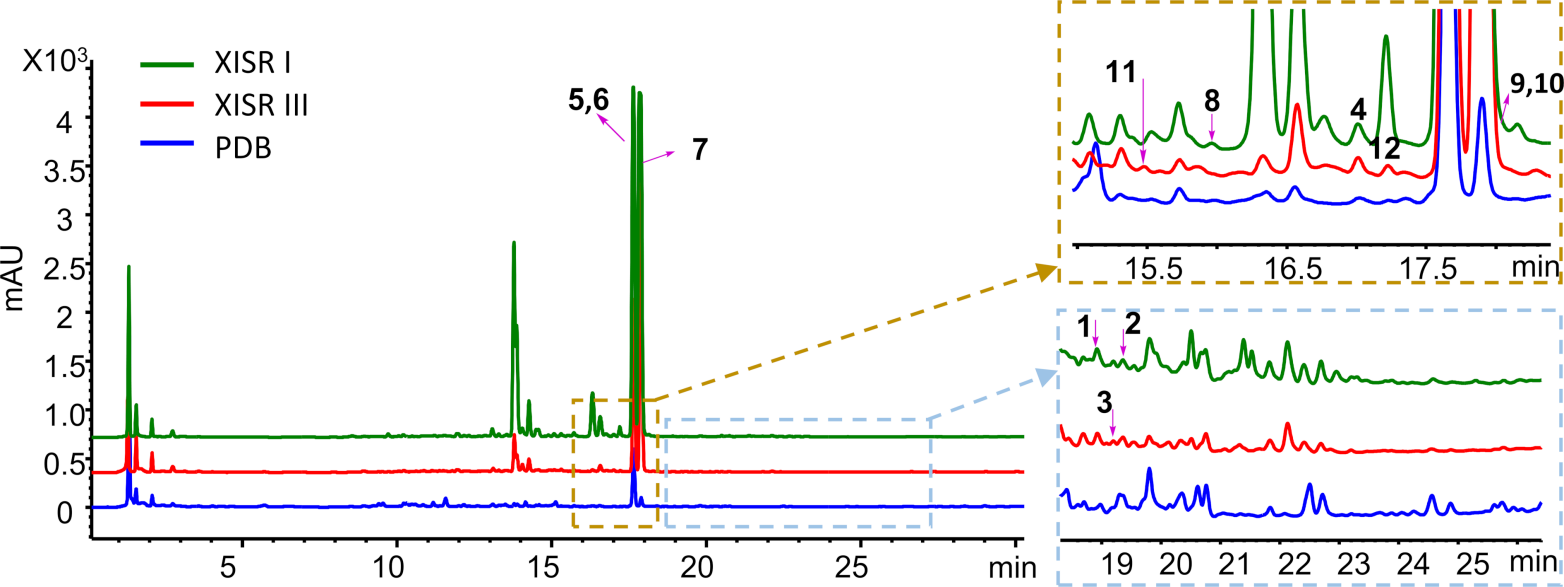
HPLC analysis of small-scale fermentation with different media. More details of media, XISR I and XISR III can be found in the methods section.

In the present study, we focused on a newly identified *Penicillium* strain, *Penicillium shentong* XL-F41. To activate the BGCs of this strain, we employed a combination of elicitors in our fermentation media, including histone deacetylase inhibitors and DNA methyltransferase inhibitors. We developed two specialized media, XISR I and XISR III, which outperformed the traditional potato dextrose broth (PDB) in stimulating the production of a greater number of metabolite peaks, as shown in Figure 1. Scaled-up fermentation allowed us to isolate and characterize two new indole terpene alkaloids, shentonins A and B (**1** and **2**), a new fatty acid **3**, and nine previously identified compounds **4**–**12**, among which were gram quantities of curvularin analogs. Our bioactivity assays identified one compound, **12**, with promising antimicrobial properties. Subsequent genome sequencing analysis pinpointed the likely BGCs associated with our isolated compounds and suggested a vast potential for the production of additional compounds, given the application of suitable activation techniques.

## Results and Discussion

### Compound isolation and structure elucidation

To activate the silent BGCs in *Penicillium shentong* XL-F41, we conducted small-scale fermentations using various media. Analysis revealed that HPLC peaks, which correspond to fermentation products, showed a lower number and abundance in the PDB medium than in the XISR I and XISR III media, as illustrated in Figure 1. Consequently, we chose XISR I and XISR III media for further fermentation.

**Figure 2:**
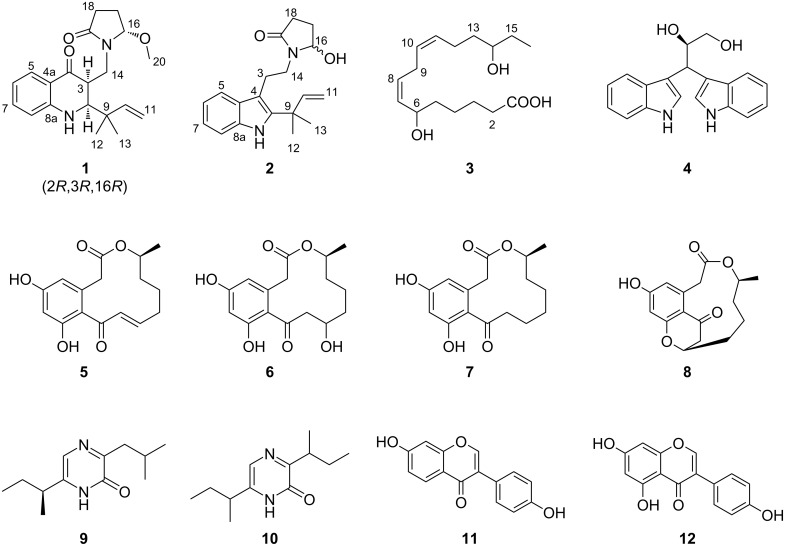
Chemical structures of compounds **1–12**.

The fermentation broth was exhaustively extracted with ethanol, after which the ethanol extract was partitioned between EtOAc and H_2_O. The EtOAc fraction was chromatographed repeatedly over silica gel and reversed-phase high-performance liquid chromatography (RP-HPLC), resulting in the isolation of pure compounds **1**–**12** (Figure 2). According to literature reports of known compounds, some of them were identified as fusarindoles B (**4**) [15], dehydrocurvularin (**5**) [16], hydroxycurvularin (**6**) [17], curvularin (**7**) [18], curvulopyran (**8**) [19], (*S*)-6-(*sec*-butyl)-3-isobutylpyrazin-2(1*H*)-one (**9**) [20], 3,6-di-*sec*-butyl-2(1*H*)-pyrazinone (**10**) [20], daidzein (**11**) [21], and genistein (**12**) [22]. Notably, compound **7**, corresponding to the major peak in our optimized fermentation (Figure 1), was obtained at the gram level.

Compound **1** (shentonin A) was obtained as a light green solid with a chemical formula of C_20_H_26_N_2_O_3_, as determined by HRMS *m*/*z* 365.1828 [M + Na]^+^ (calcd for C_20_H_26_N_2_O_3_Na^+^, 365.1835) and HRMS *m*/*z* 341.1862 [M − H]^−^ (calcd for C_20_H_25_N_2_O_3_, 341.1870). Spectroscopic analysis, including ^1^H NMR, ^13^C NMR (Table 1), and DEPT, revealed that compound **1** contains three methyl groups, one of which is oxygenated, four methines, three saturated non-protonated carbons, and two ketone carbonyl carbons (δ_C_ 175.94, δ_C_ 194.36). Its NMR data closely resemble those of brocaeloid D [23], with the notable addition of a methoxy group (δ_H_ 3.20/δ_C_ 53.92). HMBC correlations confirmed the presence of a reversed prenyl group and differentiated compound **1** from brocaeloid D by the substitution of a succinimide substructure at C-14 with a methine at C-16, indicated by the methoxy group. The position of the methoxy substituent was established by HMBC correlations, and the ^13^C NMR data suggested that compound **1** includes a 4-oxo-2,3-dihydro-(1*H*)-quinolin-3-yl fragment. The planar structure was established from HMBC correlations linking three different fragments.

**Table 1:** ^1^H and ^13^C data of compound **1** (recorded in CDCl_3_).

	δ_H_ mult (*J* in Hz)	δ_C_ mult

1	4.66 (s)	NH
2	3.09 (dd, 3.9, 1.0)	61.4, CH
3	2.92 (t, 7.6)	45.2, CH
4	–	194.3, qC
4a	–	116.9, qC
5	7.68 (dd, 7.9, 1.6)	127.2, CH
6	6.61, m	114.9, CH
7	7.28 (d, 1.7)	136.0, CH
8	6.61, m	116.7, CH
8a	–	149.9, qC
9	–	43.1, qC
10	5.65 (dd, 17.5, 10.8)	144.3, CH
11	5.02, m	114.3, CH_2_
12	0.97 (d, 7.2)	23.2, CH_3_
13	0.97 (d, 7.2)	23.5, CH_3_
14a	3.16, m	41.8, CH_2_
14b	3.90 (dd, 13.9, 8.8)	–
15	–	N
16	4.77 (dd, 6.1, 1.2)	89.7, CH
17a	1.99 (ddd,13.5,9.5)	24.3, CH_2_
17b	2.12, m	–
18a	2.37 (ddd, 17.2, 9.9)	28.9, CH_2_
18b	2.51 (dt, 17.8, 9.2)	–
19	–	175.9, qC
20	3.20, s	53.9, CH_3_

**Figure 3:**
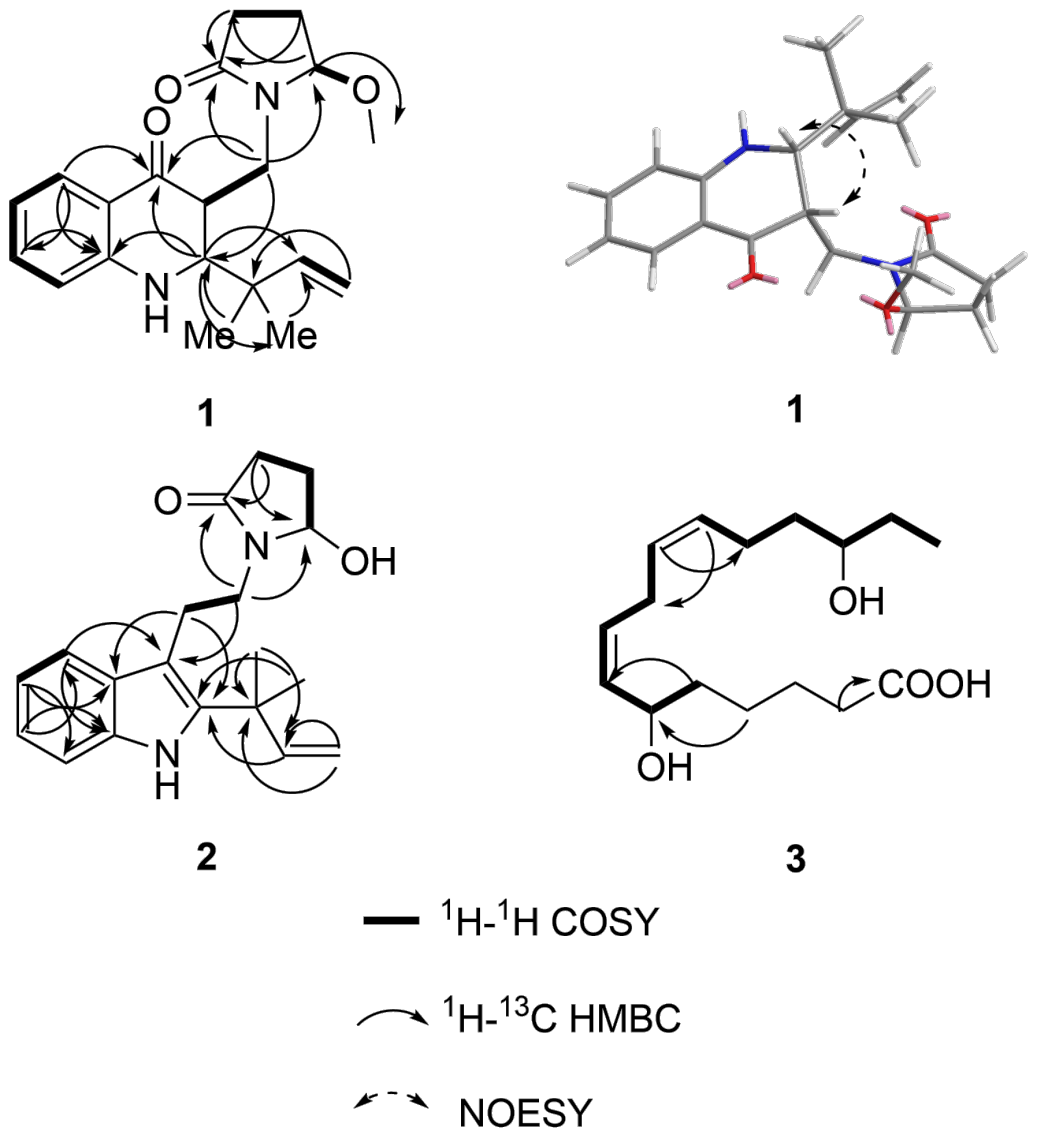
Key 2D NMR correlations of compounds **1–3**.

**Figure 4:**
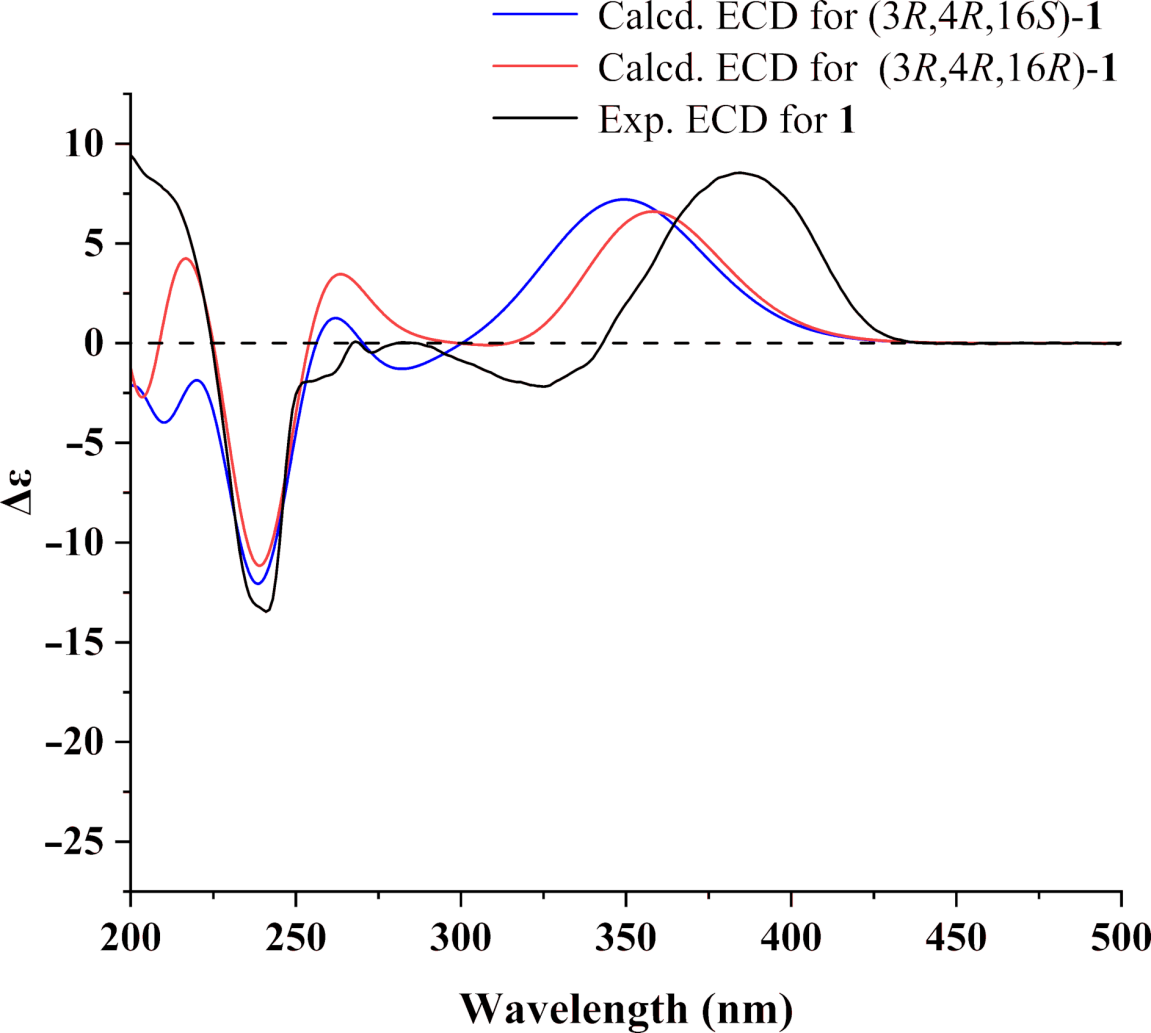
Experimental and calculated ECD spectra at the CAM-B3LYP/6-311G(d,p) level of theory for compound **1**.

Compound **1** features three stereogenic centers at C-2, C-3, and C-16. The relative configuration of C-2 and C-3 was determined as (2R*,3R*) by ^1^H-^1^H NOESY correlations (Figure 3), while the relative configuration of C-16 remains unresolved due to the inapplicability of the NOESY experiment. To establish the absolute configuration of compound **1**, electron circular dichroism (ECD) calculations were conducted using the time-dependent density functional theory (TDDFT) approach at a B3LYP/6-311G (d,p) (IEFPCM) level (Figure 4). Considering the uncertainty of the relative configuration of C-16, both ECD spectra of 2*R*,3*R,*16*R*-**1** and 2*R*,3*R,*16*S*-**1** were calculated, and compared with experimental ECD. Both calculated spectra displayed almost identical curves compared to the experimental one, which was suggestive of the 2*R*,3*R* absolute configuration for compound **1**. The calculated spectrum of 2*R*,3*R*,16*R*-**1** matched better with the experimental one, so the absolute configuration of **1** was tentatively determined to be 2*R*,3*R,*16*R*, namely shentonin A.

Compound **2** (shentonin B) was isolated as a light green solid. Its chemical formula, C_19_H_24_N_2_O_2_, was confirmed by HRMS with *m*/*z* 335.1719 [M + Na]^+^ (calcd for C_19_H_24_N_2_O_2_Na^+^, 335.1730) and *m*/*z* 311.1755 [M − H]^−^ (calcd for C_19_H_23_N_2_O_2_, 311.1765). Spectroscopic analysis using ^1^H NMR, ^13^C NMR, and DEPT (Table 2) indicated that compound **2** comprises two methyl groups, five methines, five saturated non-protonated carbons, and one ketone carbonyl carbon (δ_C_ 174.6). Its NMR profile is similar to brocaeloid C [24], with the distinction of an added succinimide substructure at N-15, where the ketone carbonyl carbon at C-16 is replaced by a hydroxy carbon. The isoprene group is consistent with that in compound **1**. HMBC cross-peaks from H-10 to C-2 and H-12 to C2 connect the indole and isoprene units, while HMBC correlations from H-14 to C-4, C-16, and C-19, and from H-3 to C-4a and C-9, elucidate the connectivity of three fragments. These data collectively establish the planar structure of compound **2**.

**Table 2:** ^1^H and ^13^C NMR data of compound **2** (recorded in CDCl_3_).

	δ_H_ mult (*J* in Hz)	δ_C_ mult

1	7.90, s	NH
2	–	140.0, qC
3	3.09 (ddd, 14.1, 9.3, 5.6)	23.9, CH_2_
3a	3.16 (ddd, 14.1, 9.5, 6.5)	–
4	–	108.1, qC
4a	–	129.6, qC
5	7.61 (dp, 7.8, 0.7)	118.3, CH
6	7.09 (ddd, 8.1, 7.0, 1.1)	119.6, CH
7	7.14 (ddd, 8.1, 7.0, 1.2)	121.7, CH
8	7.29 (dt, 8.0, 1.0)	110.6, CH
8a	–	134.2, qC
9	–	39.1, qC
10	6.14 (dd, 17.4, 10.5)	146.0, CH
11	5.16 (d, 1.1)	112.2, CH_2_
11a	5.18 (dd, 2.5, 1.1)	–
12	1.56 (d, 1.5)	27.7, CH_3_
13	–	27.8, CH_3_
14	3.51 (m)	41.5, CH_2_
14a	3.67 (ddd, 13.7, 9.5, 5.6)	–
15	–	N
16	4.98 (s)	84.3, CH
17	2.30 (ddd, 17.1, 10.1, 4.3)	28.9, CH_2_
17a	2.54 (ddd, 16.9, 9.7, 7.2)	–
18	1.76 (dddd, 13.8, 9.7, 4.3, 2.4)	28.7, CH_2_
18a	2.19 (dddd, 13.7, 10.1, 7.3, 6.4)	–
19	–	174.6, qC

Compound **3** was isolated as a transparent oily liquid, and its chemical formula, C_16_H_28_O_4_, was confirmed by LC–MS with *m*/*z* 283.2 [M − H]^−^ (calcd for C_16_H_27_O_4_, 283.2) (Figure S25 in Supporting Information File 1). Spectroscopic analyses, including ^1^H NMR, ^13^C NMR, DEPT, HSQC, COSY, and HMBC (Table 3, Figure 3), identified compound **3** as a sixteen-carbon fatty acid. Notably, two methylene carbons overlapped in the ^13^C NMR spectrum. The COSY correlations facilitated the determination of the carbon chain fragments from C-11 to C-16 and C-2 to C-10, despite two methylene signals overlapping. The carboxyl group's position at C-1 was confirmed by HMBC correlations from H-2/3. Furthermore, HMBC cross-peaks from H-12 to C-10, H-11 to C-9, and H-10 to C-12 indicated that the fragments are connected through C-11 and C-10, establishing the structure of compound **3**.

**Table 3:** ^1^H and ^13^C NMR data of compound **3** (recorded in CDCl_3_).

	δ_H_ mult (*J* in Hz)	δ_C_ mult

1	–	177.2, qC
2	2.41, m; 1.96 (dp, 12.9, 9.3)	29.3, CH_2_
3	2.57, m	29.0, CH_2_
4	2.57, m	29.0, CH_2_
5	2.41, m; 1.96 (dp, 12.9, 9.3)	29.3, CH_2_
6	5.30, m	76.4, CH
7	5.47, m	127.5, CH
8	5.66 (dt, 11.0, 7.5)	134.0, CH
9	2.86 (dt, 15.3, 7.3)	26.3, CH_2_
9a	2.96 (dt, 13.5, 7.6)	–
10	5.36, m	126.9, CH
11	5.47, m	131.0, CH
12	2.19 (q, 7.6)	23.7, CH_2_
13	1.49, m	36.5, CH_2_
14	3.53 (tt, 8.3, 4.4)	72.8, CH
15	1.49, m	30.5, CH_2_
16	0.94 (t, 7.5)	10.0, CH_3_

### Biological activities

In our bioassays, we evaluated the inhibitory activity of all isolated compounds against a panel of microorganisms (Table 4), including *Escherichia coli* (ATCC 25922), *Candida albicans* (ATCC 76485), *Staphylococcus aureus* (ATCC 27154), *Pseudomonas fulva* (CGMCC 1.15147), and *Enterobacter hormaechei* (CGMCC 1.10608). The results indicated that compounds **3**, **5**, **6**, **7**, and **12** were active against *Candida albicans*. Notably, compound **12** showed particularly promising inhibitory activity against this fungal pathogen.

**Table 4:** Antimicrobial activity of compounds **1**–**12**. Minimum inhibitory concentrations were shown in µg/mL.

No.	*Escherichia coli*	*Candida albicans*	*Staphylococcus aureus*	*Pseudomonas Fulva*	*Enterobacter hormaechei*

**1**	–	–	>100	–	–
**3**	–	50–100	–	>100	>100
**4**	–	>100	–	–	–
**5**	>100	25–50	>100	>100	>100
**6**	>100	25–50	>100	>100	>100
**7**	>100	64–128	>100	>100	>100
**8**	–	–	–	>100	>100
**9**	>100	>100	–	>100	>100
**11**	–	–	–	–	–
**12**	>100	12.5–25	>100	>100	>100

### Genome sequencing analysis

The genome sequencing yielded 7,118,236 reads with an average read length of 1,858.7 bp. The assembled genome is 34,621,366 bp long, comprising 9 contigs with a mean contig length of 3,846,818.44 bp, and the longest contig is 5,975,444 bp. The genome's GC content is 46.43%. Annotation of the genome sequence of *Penicillium shentong* XL-F41 identified 11,235 coding sequences and 172 tRNA genes.

Upon utilizing the fungal version of antiSMASH 7.0 [25] software for the analysis of the *Penicillium shentong* XL-F41 genome, we identified 46 BGCs. These include 13 NRPS-like fragments, 6 NRPS, 13 type I PKS, 2 PKS/NRPS hybrids, 1 NI-siderophore, 2 NRP-metallophore/NRPS hybrids, 1 fungal RiPP with POP or UstH peptidase types, 1 fungal-RiPP-like/T1PKS, 1 betalactone, 1 PKS type I/NRPS/indole hybrid, 1 fungal-RiPP-like/T1PKS hybrid, 1 NRP-metallophore/NRPS hybrid, NRPS-like/terpene/phosphonate hybrids, 3 terpenes, and 1 indole-related cluster (Table 5).

**Table 5:** Biosynthetic gene clusters of the *Penicillium shentong* XL-F41.

BGC	Type	Putative product

1.1	NRPS-like	
1.2	NRPS-like	
1.3	NI-siderophore	
1.4	NPR-metallophore, NRPS	
1.5	NRPS	
1.6	PKS type I	
1.7	PKS type I	
1.8	PKS type I	
1.9	PKS type I	
1.10	NRPS-like	
2.1	NRPS-like	
2.2	NRPS-like	
2.3	PKS type I	
2.4	NRPS-like	
3.1	PKS type I	
3.2	terpene	
3.3	fungal-RiPP	
3.4	PKS type I	
3.5	PKS type I	
4.1	PKS type I	
4.2	NRPS-like	
4.3	NRPS	
4.4	NRPS-like	
5.1	PKS type I, NRPS	
5.2	NRPS-like	
5.3	PKS type I, NRPS, indole	
5.4	PKS type I	
5.5	betalactone	
6.1	NRPS	
6.2	fungal-RiPP-like, T1PKS	
6.3	PKS type I	
6.4	terpene	
6.5	NRP-metallophore, NRPS	
7.1	NRPS	
7.2	NRPS-like, terpene, phosphonate	
7.3	indole	shentonins A and B
7.4	terpene	
7.5	NRPS-like	
8.1	NRPS-like	
8.2	NRPS, PKS type I	
9.1	PKS type I	dehydrocurvularin
9.2	NRPS	
9.3	NRPS-like	
9.4	NRPS	
9.5	NRPS-like	
9.6	PKS type I	

**Figure 5:**
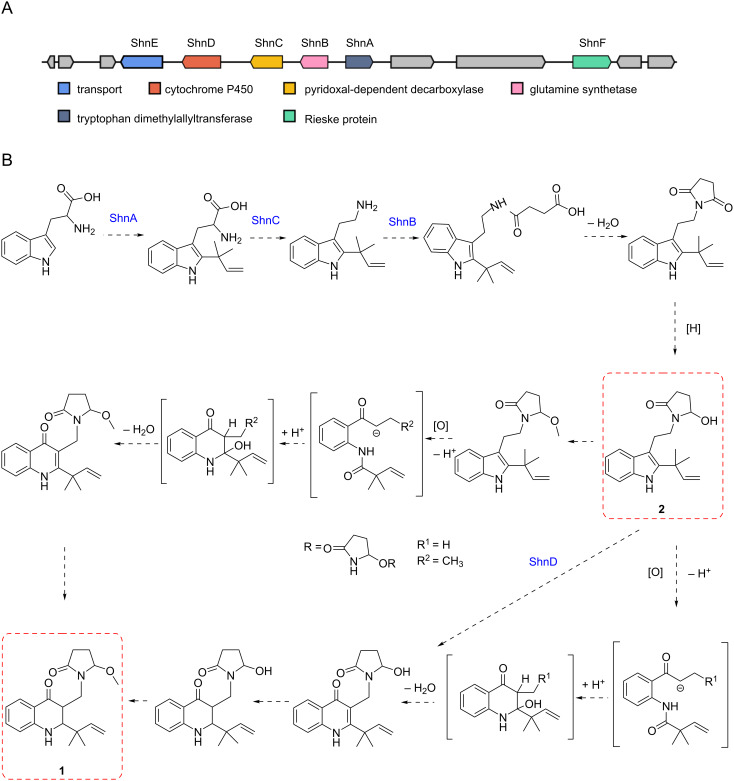
Biosynthetic exploration of compounds **1** and **2**. A: The schematic presents the biosynthetic gene cluster for compounds **1** and **2**, highlighting ShnA, ShnB, ShnC, ShnD, and ShnE as core genes. B: The diagram proposes biosynthetic pathways for compounds **1** and **2**, detailing three potential mechanisms that could convert the five-membered ring structure of compound **2** into the six-membered ring structure of compound **1**.

BGC 7.3, identified as an indole-type gene cluster, includes genes for cytochrome P450, pyridoxal-dependent decarboxylase, glutamine synthase, and tryptophan dimethyltransferase (Figure 5). These genes are likely crucial for the biosynthesis of the newly isolated alkaloids, **1** and **2**. In examining the XL-F41 genome for methyltransferase domain-containing BGCs, we found a methyltransferase near BGC 7.3, suggesting its involvement in adding a methoxy group at the C16 position of compound **1**. From these key enzyme genes, we propose a hypothetical biosynthetic pathway (Figure 5).

Compounds **1** and **2** are hypothesized to be synthesized from a tryptophan precursor via a shared biosynthetic pathway (Figure 5). Briefly, the prenyl group is attached to tryptophan through a prenylation reaction catalyzed by ShnA, followed by the decarboxylation of the carboxy group by ShnC. Subsequently, compound **2** is formed by the addition of succinimide to N15 in **1** via a reaction catalyzed by ShnB. The transformation of the five-membered pyrrole ring in compound **2** to the six-membered ring in compound **1** is particularly intriguing. For this transformation, three hypotheses are considered. One suggests that the methyl modification at the oxygen atom of the succinimide ring occurs first, which is then followed by a ring-opening rearrangement. Alternatively, it is proposed that the ring-opening rearrangement precedes the methyl modification at the oxygen atom of the succinimide ring.

We aim to confirm the initial step of this pathway, where tryptophan and DMAPP are catalyzed by the enzyme ShnA to form a reverse prenylated tryptophan. However, attempts to express the protein in various *Escherichia coli* hosts were unsuccessful, suggesting that eukaryotic hosts might be more suitable for future studies. We plan to conduct further experiments to substantiate the hypothesis regarding the biosynthetic pathways in the future.

## Conclusion

In the present study, we fermented *Penicillium shentong* XL-F41 by adding a series of elicitors in the medium, which led us to identify twelve compounds, including two new indole alkaloids, shentonins A and B (**1** and **2**), and a new fatty acid (**3**).

Notably, compound **1** differs from the known brocaeloid D by the addition of a methyl group, and there is a change in the relative stereochemistry at C2 and C3. In addition, the conversion of the five-membered pyrrole ring in compound **2** to the six-membered piperidine ring in compound **1** is intriguing.

Although there have been many reports on the biosynthetic pathways from tryptophan to quinoline rings. Through our analysis, we discovered that tryptophan in the primary metabolic pathway is primarily catalyzed by indoleamine 2,3-dioxygenase and tryptophan 2,3-dioxygenase (IDO and TDO), as well as canine urinary tryptophan 3-monooxygenase, to form quinoline rings [26]. Quinine is frequently cited as one of the primary forms of quinoline rings in secondary metabolic pathways. Francesco Trenti et al. [27] studied some of the biosynthesis processes of quinine, in which enzymes involved are much more complex than primary metabolism, such as medium-chain alcohol dehydrogenase (*Cp*DCS), esterase (*Cp*DCE), P450 and O-methyltransferase (*Cp*OMT1) (Figure S37). Through genome excavation and analysis of *Penicillium shentong* XL-F41, a significant difference was discovered between the key enzymes involved in the formation of product compound **1** and those previously reported. This suggests that the formation of the quinoline ring in compound **1** may represent a new and unreported biosynthetic pathway.

Moreover, to address the low yields that hindered the determination of absolute stereochemistry, we attempted to boost the production of compounds **1** and **2** by supplementing the medium with the precursor tryptophan. Contrary to our expectations, this approach did not increase their production. Our next step is to plan the heterologous expression of core genes in proper fungal hosts to improve production and investigate the biosynthetic pathways of compounds **1** and **2**.

Furthermore, we conducted a genome sequencing analysis of *Penicillium shentong* XL-F41, which allowed us to pinpoint the biosynthetic gene clusters (BGCs) associated with our isolated compounds and reveal the biosynthetic capabilities of this strain. Despite the addition of various elicitors to the fermentation medium, numerous BGCs remain uncharacterized, indicating that additional strategies are required to fully elucidate the compounds encoded by all BGCs.

## Experimental

### General experimental procedures

HRESIMS spectra were acquired using a Waters ACQUITY UPLC I-Class-Vion IMS Q-Tof liquid chromatograph mass spectrometer. For NMR analysis, we utilized an AVANCE II 600 spectrometer, referencing residual solvent peaks at δ_H_/δ_C_ 7.27/77.0 ppm in CDCl_3_ for chemical shift calibration. We utilized commercial silica gel from Yantai Xinnuo New Material Technology Co., Ltd, Yantai, China, available in 100–200 and 200–300 mesh sizes. Reversed-phase HPLC analyses were conducted on an Agilent 1260 instrument equipped with a DAD detector and an Agilent ZORBAX SB-C18 column (5 µm, 4.6 × 150 mm). The solvents used for HPLC were supplied by Yantai Huisente New Material Technology Co., Ltd, Yantai, China.

### Fungus isolation and characterization

The fungus *Penicillium shentong* XL-F41 was isolated from soil collected in Shentong Mountain, Shandong Province, China, in June 2022. It was cultured on potato dextrose agar (PDA) at 28 °C. Sequencing and comparison with the GenBank database confirmed its identification as *Penicillium* sp. This strain is preserved at the Shandong Laboratory of Yantai Drug Discovery.

### Fermentation in shaking flasks

For large-scale fermentations, fresh mycelia of *Penicillium shentong* XL-F41 were first cultivated in liquid potato dextrose broth at 28 °C for 2 days. Subsequently, they were inoculated into XISR I and XISR III liquid media (total volume 30 L) with a 20% inoculum dose. The cultures were further fermented for 14 days at 28 °C and 200 rpm.

#### Media recipes

XISR I medium (yeast extract 4 g/L; malt extract 10 g/L; glucose 4 g/L; MgCl_2_ 1 µM; FeSO_4_ 1 µM; KI 2 g/L, KCl 2 g/L, KBr 2 g/L, NaNO_2_ 2 g/L; H_2_O_2_ 20 µM; (methyl jasmonate) MeJA 10 µM).

XISR III medium (yeast extract 4 g/L; soy flour 10 g/L; glucose 30 g/L; MgCl_2_ 1 µM; FeSO_4_ 1 µM; KI 2 g/L, KCl 2 g/L, KBr 2 g/L, NaNO_2_ 2 g/L; H_2_O_2_ 20 µM; MeJA 10 µM; 5-azacytidine 6 µM; suberoylanilide hydroxamic acid 6 µM; sodium butyrate 6 µM).

### Extraction

The mycelium was separated from the fermentation broth using a centrifuge and subsequently extracted with ethanol in a 1:1 ratio using ultrasound, three times for 20 minutes each. The combined organic solvents were dried with a rotary evaporator to yield an ethanol extract. This extract was further processed with ethyl acetate (EtOAc) three times. The combined EtOAc phase was then dried using a rotary evaporator to obtain the EtOAc extract, which was stored at −80 °C until further purification process.

The above fermentation broth was adsorbed onto macroporous resin for 4 hours or left overnight. It was then eluted with deionized water and ethanol through a chromatography column. The ethanol eluate was concentrated to dryness using a rotary evaporator. Subsequently, the ethanol extract underwent a triple extraction with EtOAc. The combined EtOAc extracts were dried using a rotary evaporator to obtain the final EtOAc extract, which was stored at −80 °C until further isolation process.

### Compounds purification

The EtOAc extract (3.5 g) obtained from the fermentation broth of XISR I medium underwent column chromatography on silica using a gradient of petroleum ether–ethyl acetate (PE–EA) and ethyl acetate–methanol (EA–MeOH) to yield 22 fractions (Fr.A–Fr.N) as determined by TLC analysis. Fraction Fr.J (62.32 mg) was further purified using silica gel column chromatography with a PE–EtOAc gradient to obtain nine subfractions (Fr.J1–Fr.J9). Subfraction Fr.J7 (17 mg) was then subjected to RP-HPLC with an acetonitrile–water gradient, resulting in the isolation of compounds **1** (1.36 mg) and **2** (0.36 mg).

Similarly, the EtOAc extract (9.3 g) from the fermentation broth of XISR III medium was fractionated by column chromatography on silica with PE–EA and EA–MeOH gradients to yield 22 fractions (Fr.1–Fr.22) based on TLC analysis. Fraction Fr.12 (200 mg) was purified using a PE–EtOAc gradient to produce seven subfractions (Fr.a–Fr.g). Subfraction Fr.e (23.19 mg) was processed using RP-HPLC with an acetonitrile–water gradient, leading to the isolation of compound **3** (2.19 mg).

#### Physical and spectroscopic data of compounds **1**–**3**

Shentonin A (**1**): light green solid; [α]_D_^20^ +40.0 (*c* 0.17, CH_3_OH); UV (CH_3_OH) λ_max_, nm (log ε): 400 (3.45), 240 (4.24) nm; IR (KBr) *ν*_max_: 3347, 2960, 2926, 1688, 1654, 1612, 1260, 1078, 1021, 797 cm^−1^; for ^1^H NMR (CDCl_3_, 600 MHz) and ^13^C NMR (CDCl_3_, 125 MHz) spectral data, see Table 1; HRESIMS (*m/z*): [M − H]^−^ calcd for 341.1870; found, 341.1862.

Shentonin B (**2**): light green solid; [α]_D_^20^ +5.3 (*c* 0.07, CH_3_OH); UV (CH_3_OH) λ_max_m nm (log ε): 280 (3.59), 220 (4.30) nm; IR (KBr) ν_max_: 3315, 2969, 1667, 1461, 1159, 1138, 1061, 981, 914, 742 cm^−1^; for ^1^H NMR (CDCl_3_, 600 MHz) and ^13^C NMR (CDCl_3_, 125 MHz) spectral data, see Table 1; HRESIMS (*m/z*): [M − H]^−^ calcd for 311.1765; found, 311.1755.

Compound **3**: transparent oily liquid; [α]_D_^20^ −46.7 (*c* 0.18, CH_3_OH); IR (KBr) ν_max_: 3432, 2960, 2923, 1762, 1450, 1260, 1180, 1016, 800 cm^−1^; for ^1^H NMR (CDCl_3_, 600 MHz) and ^13^C NMR (CDCl_3_, 125 MHz) spectral data, see Table 1; LC–MS (*m/z*): [M − H]^−^ calcd for 283.2; found, 283.2.

### Antimicrobial activity evaluation

All isolated compounds were dissolved in 1% DMSO and introduced to pathogenic bacteria or fungi in LB or PDB media. The 96-well plates were incubated at 37 °C for 18 hours, with 1% DMSO serving as the negative control. After incubation, the OD_600_ of the bacterial cultures was measured using a Microplate Reader.

## Supporting Information

File 1 NMR and mass spectra of isolated compounds.

## Data Availability

The data that supports the findings of this study is available from the corresponding author upon reasonable request.
