# Supplementary material for: Chemical and biosynthetic potential of Penicillium shentong XL-F41
Source: Beilstein J Org Chem. 2024 Mar 15;20:597–606. doi: 10.3762/bjoc.20.52 (PMC10949001; doi:10.3762/bjoc.20.52)
Supplement: File 1 — NMR and mass spectra of isolated compounds. [file Beilstein_J_Org_Chem-20-597-s001.pdf]

BEILSTEIN JOURNAL OF ORGANIC CHEMISTRY

Supporting Information

for

Chemical and biosynthetic potential of *Penicillium shentong* XL-F41

Ran Zou, Xin Li, Xiaochen Chen, Yue-Wei Guo and Baofu Xu

Beilstein J. Org. Chem. **2024,** *20,* 597–606. doi:10.3762/bjoc.20.52

NMR and mass spectra of isolated compounds

License and Terms: This is a supporting information file under the terms of the Creative Commons Attribution License (https://creativecommons.org/licenses/by/4.0). Please note that the reuse, redistribution and reproduction in particular requires that the author(s) and source are credited and that individual graphics may be subject to special legal provisions.
The license is subject to the *Beilstein Journal of Organic Chemistry* terms and conditions: (https://www.beilstein-journals.org/bjoc/terms)

Table of contents

Figure S1. ^{1}H NMR spectrum (600 MHz, chloroform-d) of **1** S3

Figure S2. ^{13}C NMR spectrum (600 MHz, chloroform-d) of **1** S3

Figure S3. HSQC spectrum (600 MHz, chloroform-d) of **1** S4

Figure S4. ^{1}H-^{1}H COSY spectrum (600 MHz, chloroform-d) of **1** S4

Figure S5. HMBC spectrum (600 MHz, chloroform-d) of **1** S5

Figure S6. NOESY spectrum (600 MHz, chloroform-d) of **1** S5

Figure S7. HRESIMS spectrum of **1** S6

Figure S8. FT-IR spectrum of compound **1** S6

Figure S9. Chemical structure of compound **1** S7

Figure S10. ^{1}H NMR spectrum (600 MHz, chloroform-d) of **2** S8

Figure S11. ^{13}C NMR spectrum (600 MHz, chloroform-d) of **2** S8

Figure S12. HSQC spectrum (600 MHz, chloroform-d) of **2** S9

Figure S13. ^{1}H-^{1}H COSY spectrum (600 MHz, chloroform-d) of **2** S9

Figure S14. HMBC spectrum (600 MHz, chloroform-d) of **2** S10

Figure S15. NOESY spectrum (600 MHz, chloroform-d) of **2** S10

Figure S16. HRESIMS spectrum of **2** S11

Figure S17. FT-IR spectrum of compound **2** S11

Figure S18. Chemcial structure of compound **2** S12

Figure S19. ^{1}H NMR spectrum (600 MHz, chloroform-d) of **3** S13

Figure S20. ^{13}C NMR spectrum (600 MHz, chloroform-d) of **3** S13

Figure S21. HSQC spectrum (600 MHz, chloroform-d) of **3** S14

Figure S22. ^{1}H-^{1}H COSY spectrum (600 MHz, chloroform-d) of **3** S14

Figure S23. HMBC spectrum (600 MHz, chloroform-d) of **3** S15

Figure S24. NOESY spectrum (600 MHz, chloroform-d) of **3** S15

Figure S25. LC-MS spectrum of **3** S16

Figure S26. FT-IR spectrum of compound **3** S16

Figure S27. Chemcial structure of compounds **3** S16

Figure S28. ^{1}H NMR spectrum (600 MHz, chloroform-d) of **4** S17

Figure S29. ^{1}H NMR spectrum (600 MHz, chloroform-d) of **5** S18

Figure S30. ^{1}H NMR spectrum (600 MHz, acetone-d_6) of **6** S19

Figure S31. ^{1}H NMR spectrum (600 MHz, acetone-d_6) of **7** S20

Figure S32. ^{1}H NMR spectrum (600 MHz, chloroform-d) of **8** S21

Figure S33. ^{1}H NMR spectrum (600 MHz, chloroform-d) of **9** S22

Figure S34. ^{1}H NMR spectrum (600 MHz, chloroform-d) of **10** S23

Figure S35. ^{1}H NMR spectrum (600 MHz, acetone-d_6) of **11** S24

Figure S36.1H NMR spectrum (600 MHz, methanol-d_4) of **12**...................... S25

Figure S37. The biosynthetic pathways of the quinoline ring formation S26

References S27

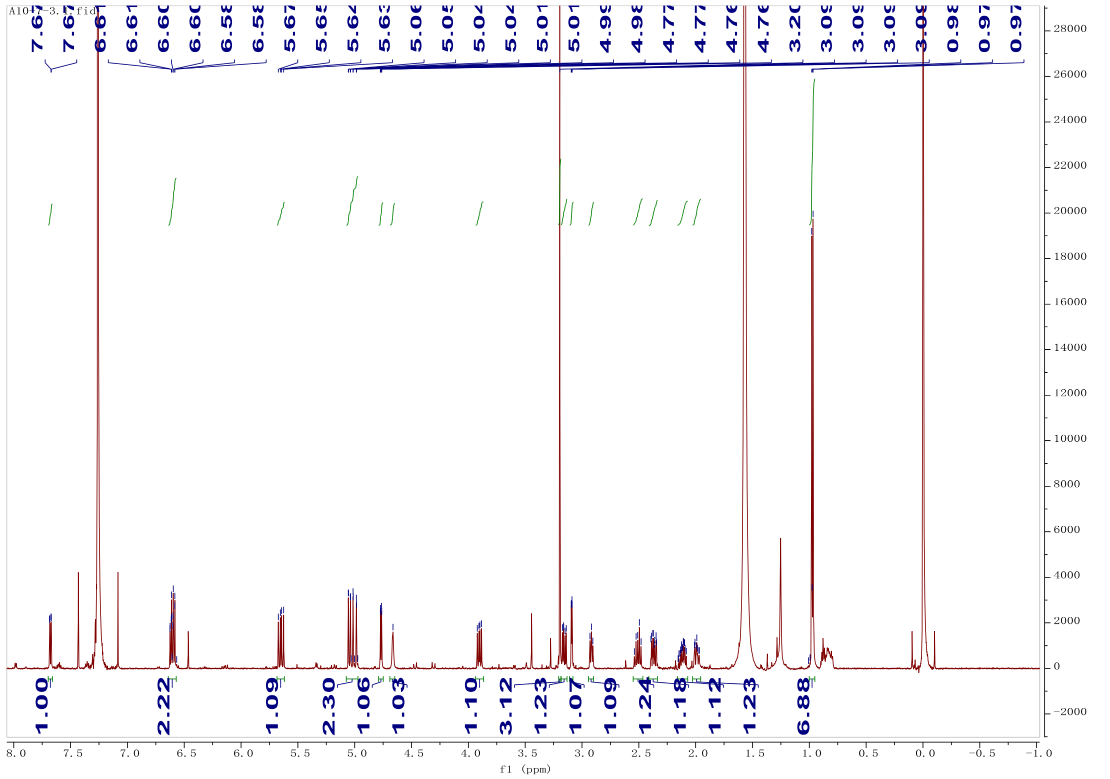

Figure S1. ^{1}H NMR spectrum (600 MHz, chloroform-d) of **1**

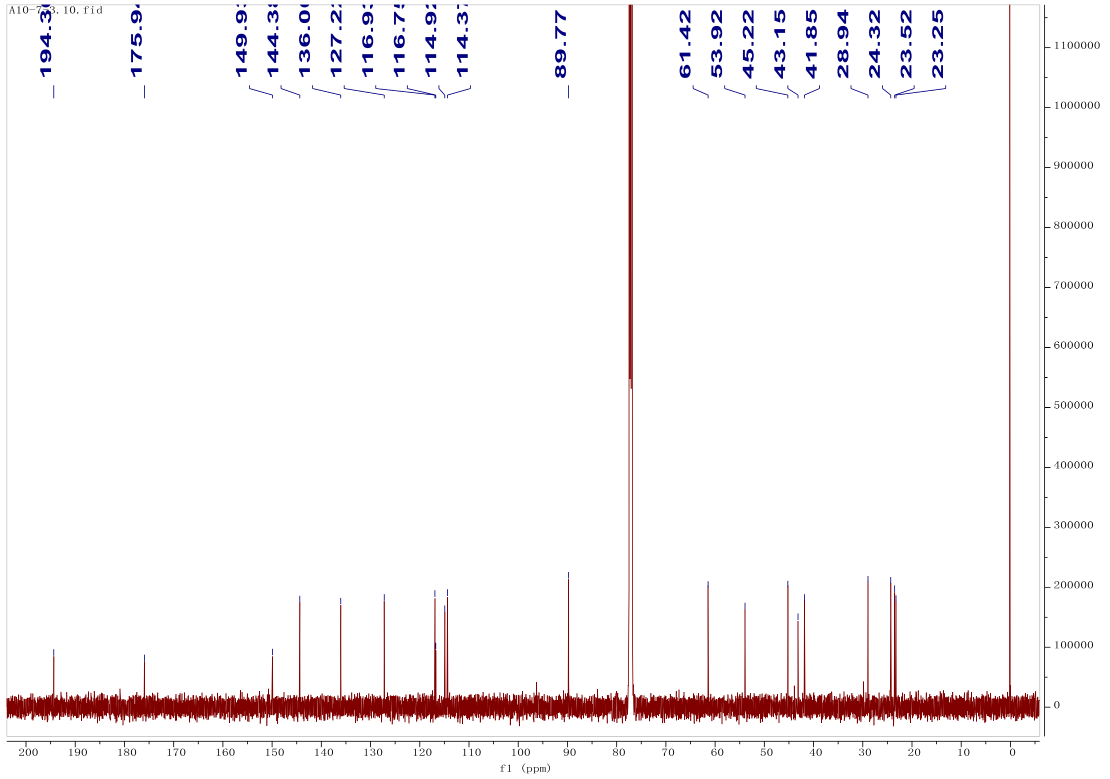

Figure S2. ^{13}C NMR spectrum (600 MHz, chloroform-d) of **1**

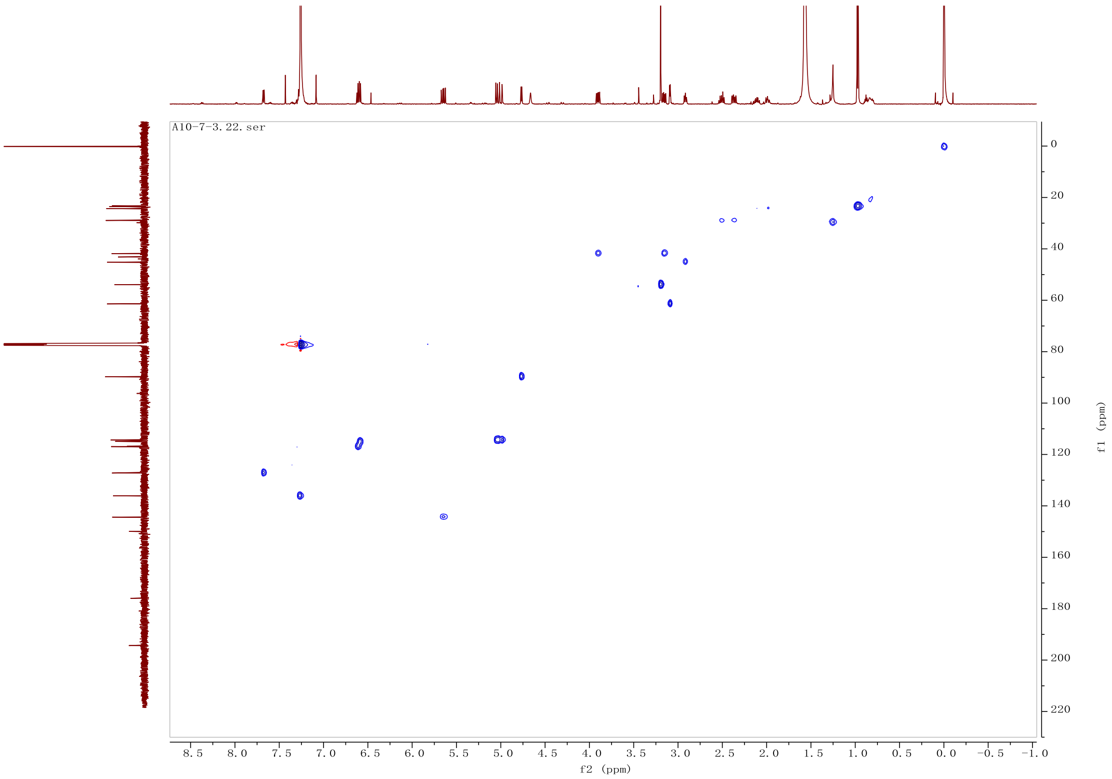

Figure S3. HSQC spectrum (600 MHz, chloroform-d) of **1**

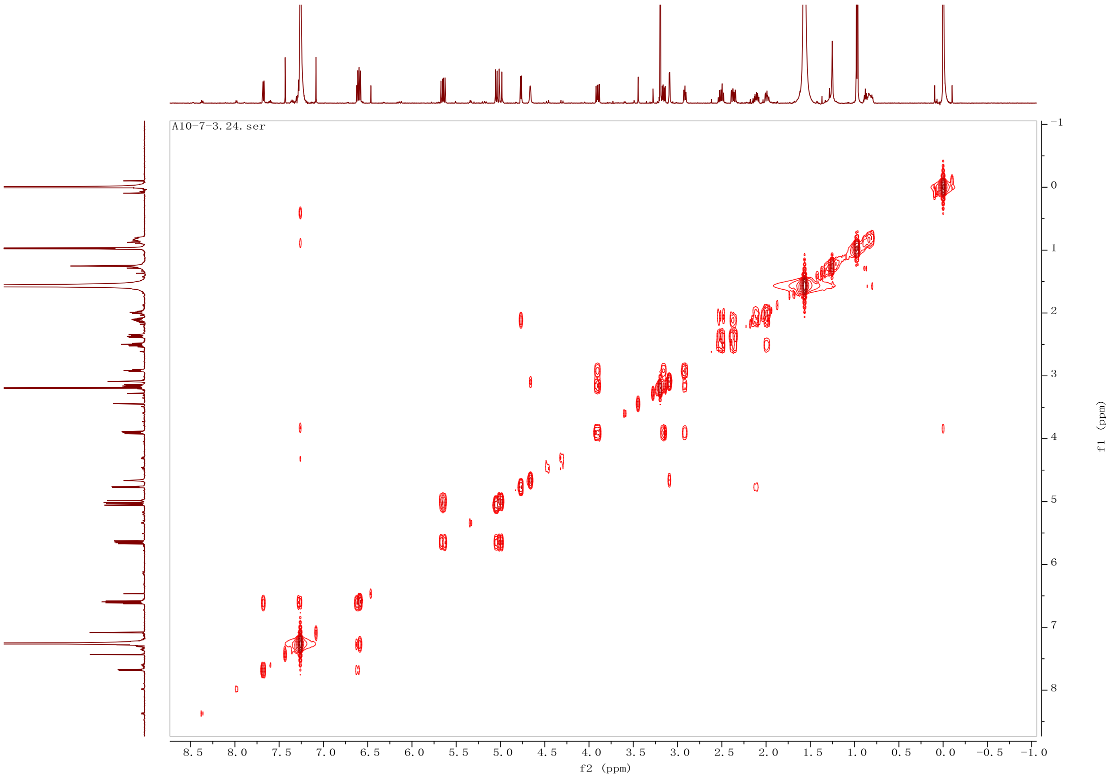

Figure S4. ^{1}H-^{1}H COSY spectrum (600 MHz, chloroform-d) of **1**

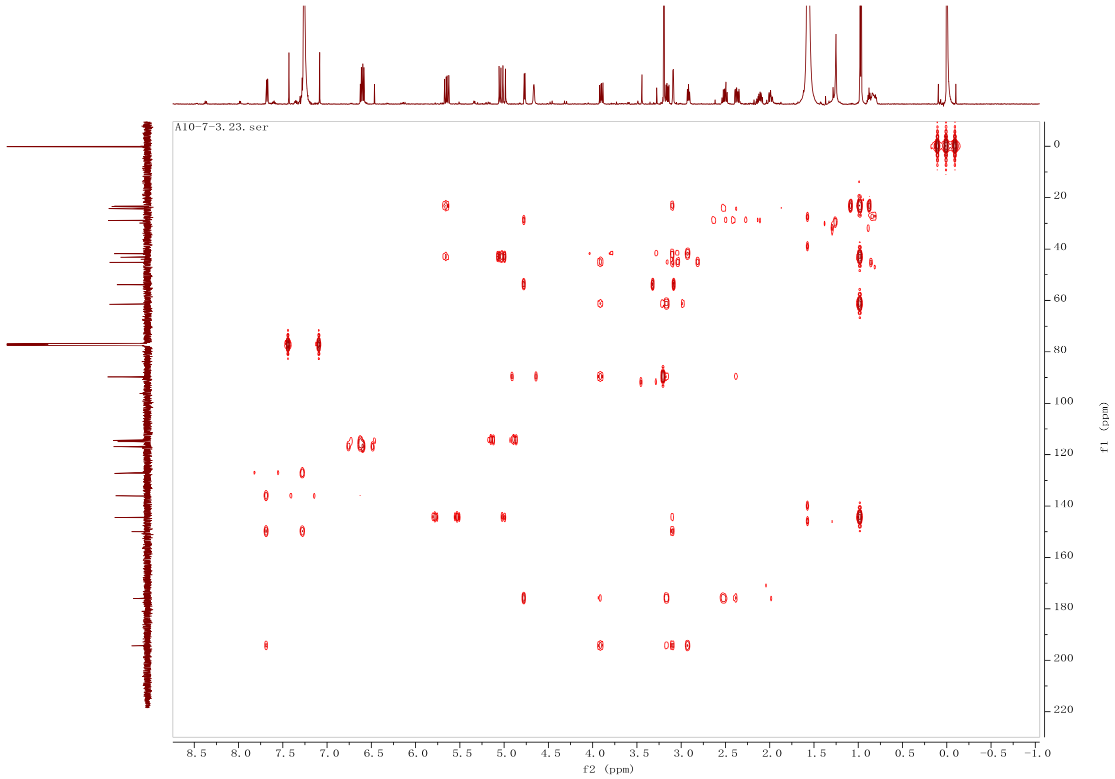

Figure S5. HMBC spectrum (600 MHz, chloroform-d) of **1**

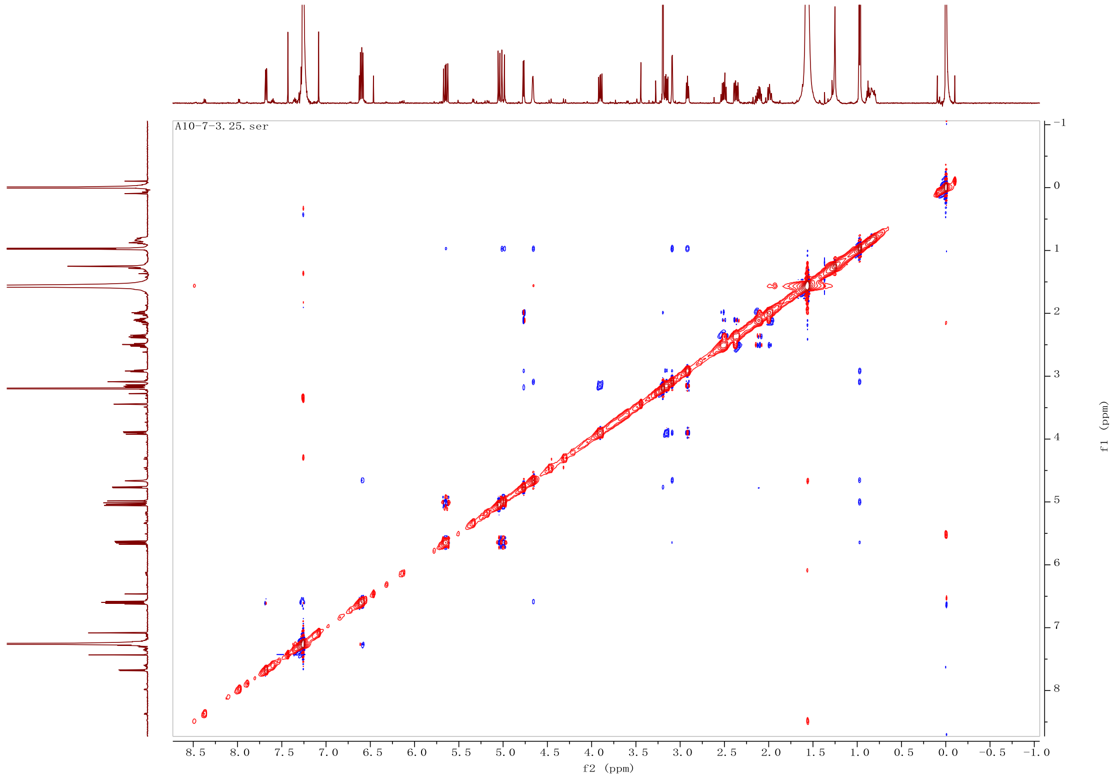

Figure S6. NOESY spectrum (600 MHz, chloroform-d) of **1**

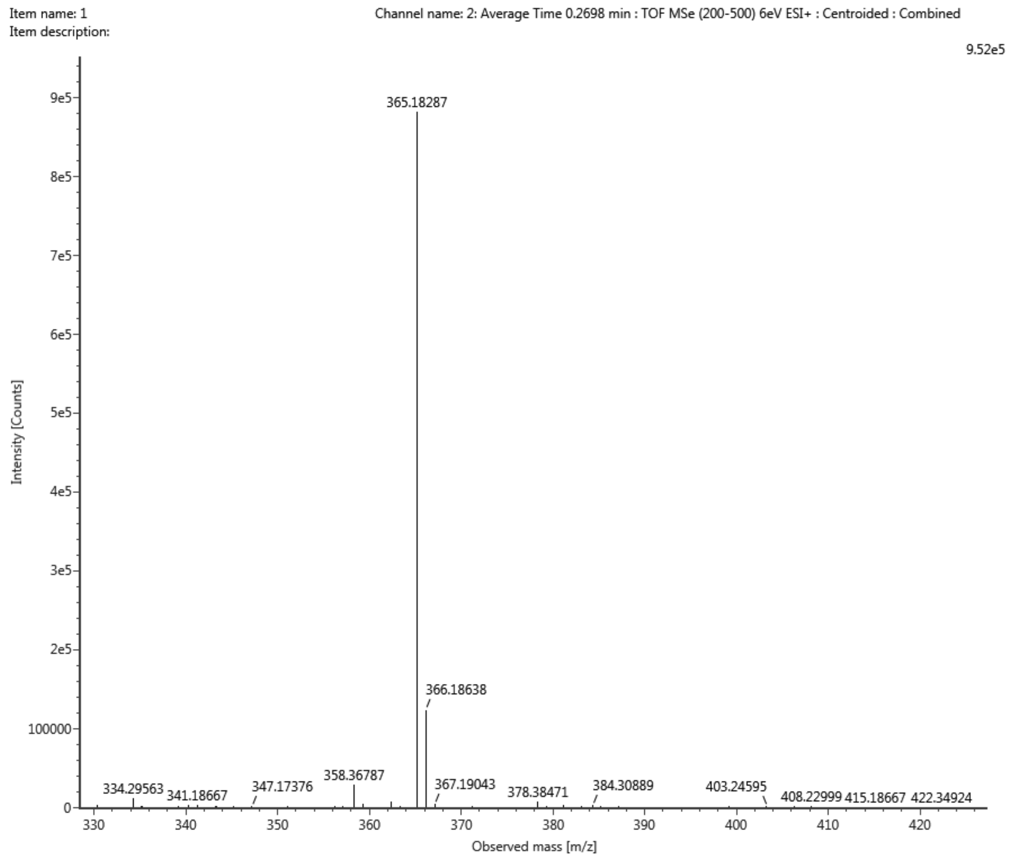

Figure S7. HRESIMS spectrum of **1**

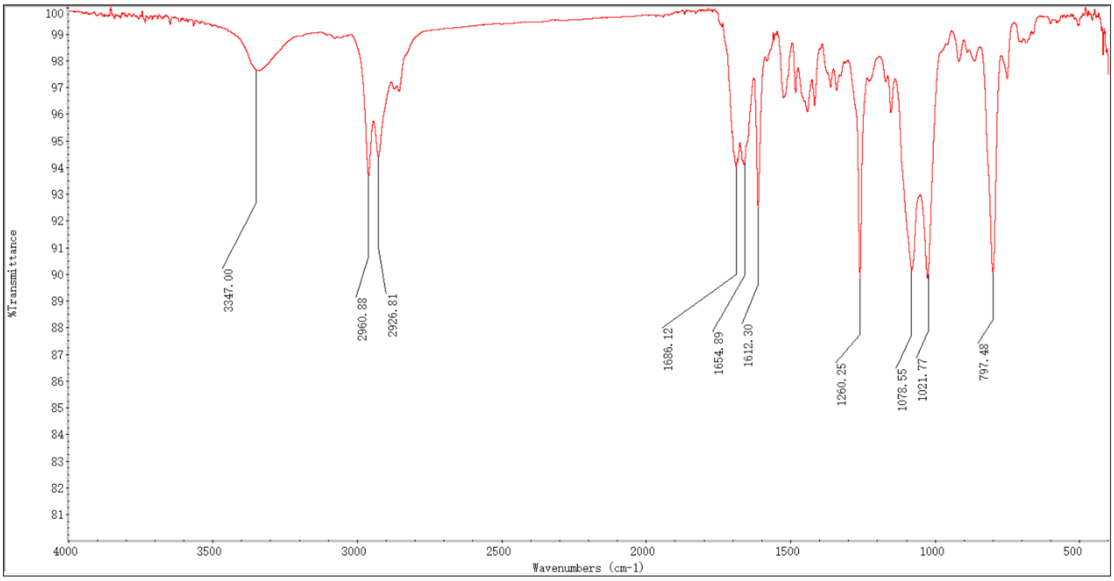

Figure S8. FT-IR spectrum of compound **1**

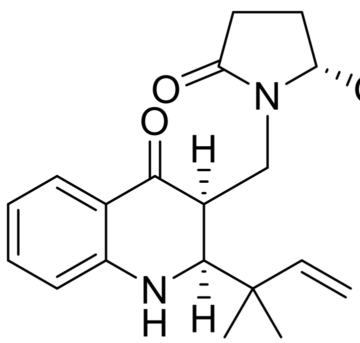

Figure S9. Chemical structure of compound **1**

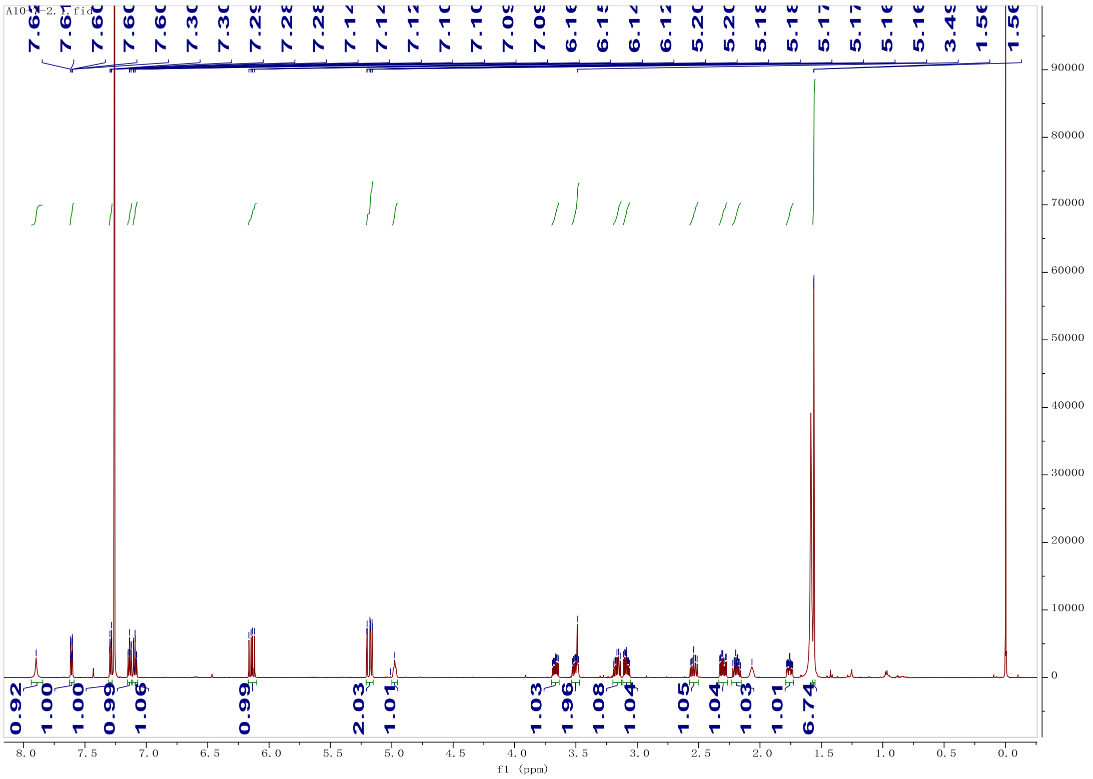

Figure S10. ^{1}H NMR spectrum (600 MHz, chloroform-d) of **2**

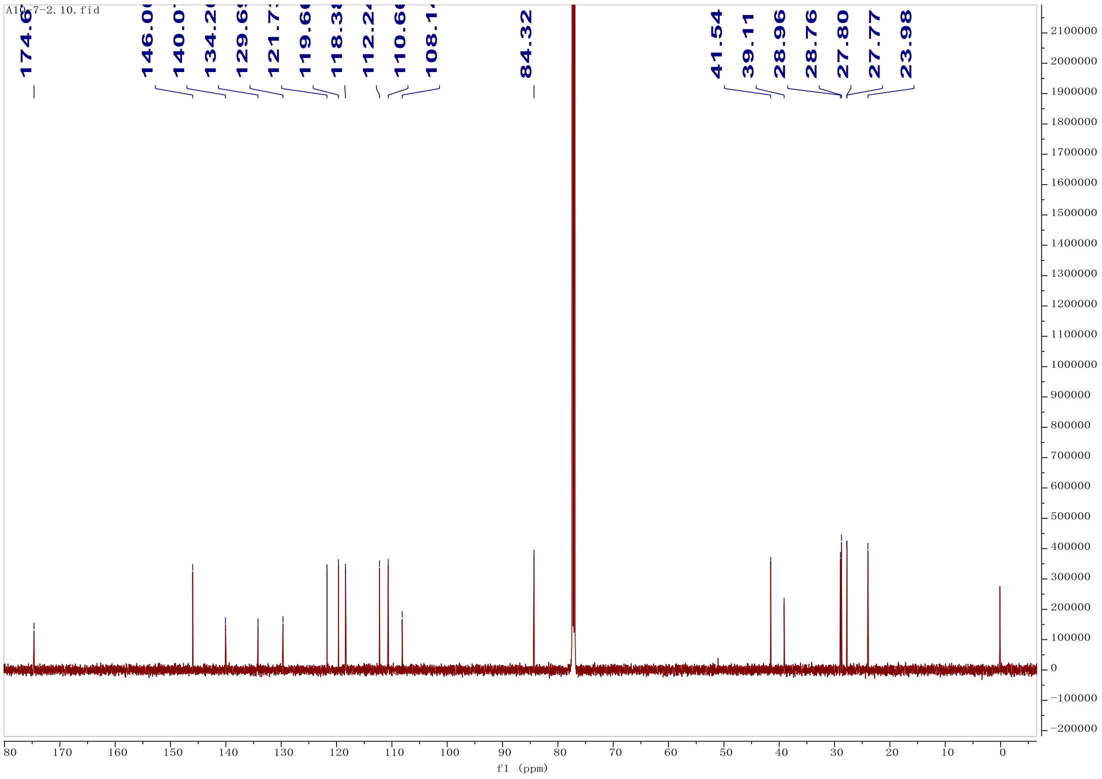

Figure S11. ^{13}C NMR spectrum (600 MHz, chloroform-d) of **2**

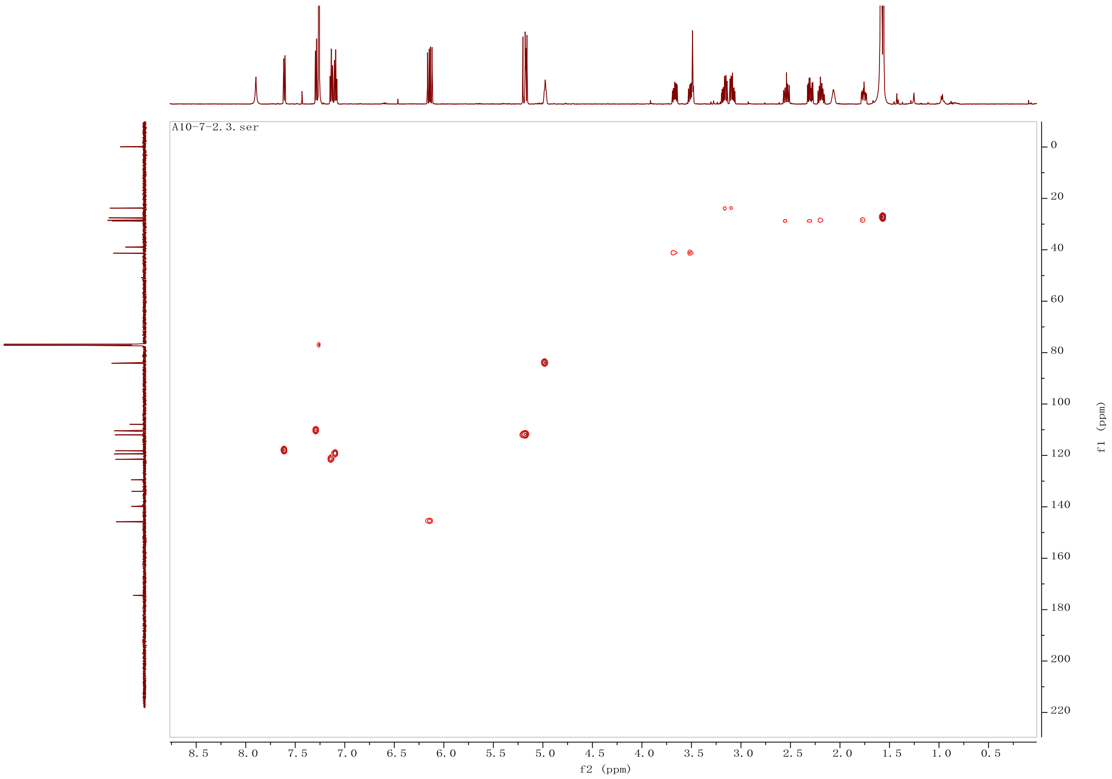

Figure S12. HSQC spectrum (600 MHz, chloroform-d) of **2**

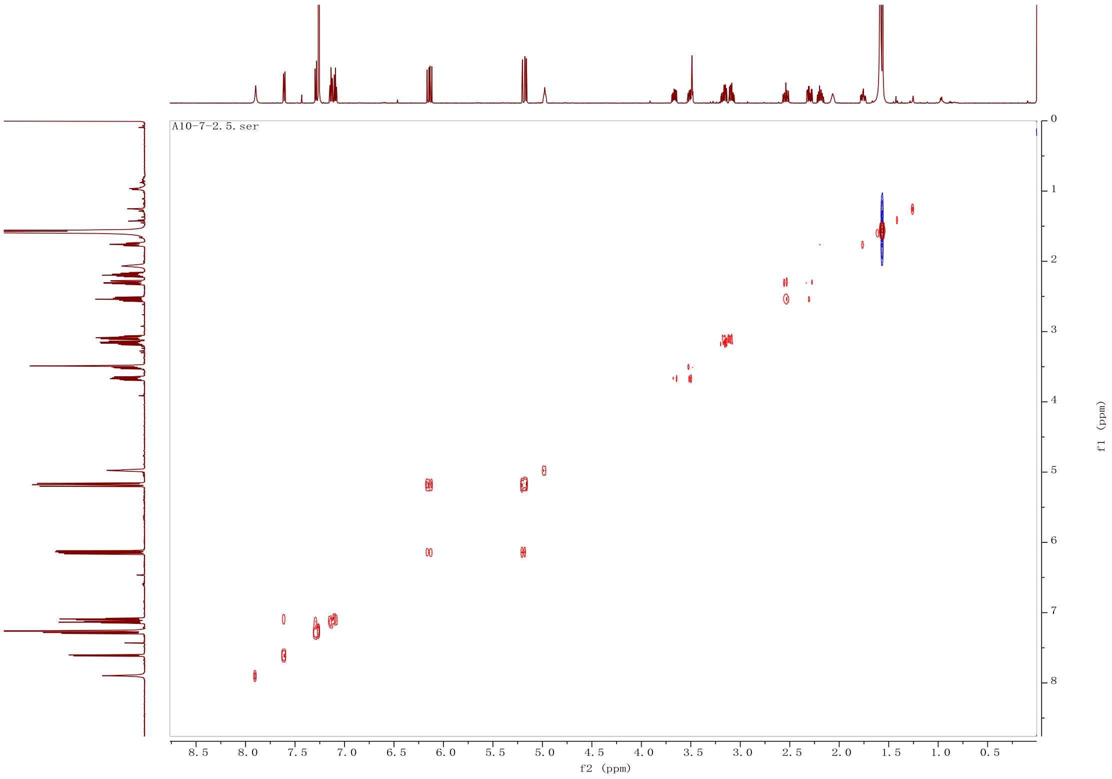

Figure S13. ^{1}H-^{1}H COSY spectrum (600 MHz, chloroform-d) of **2**

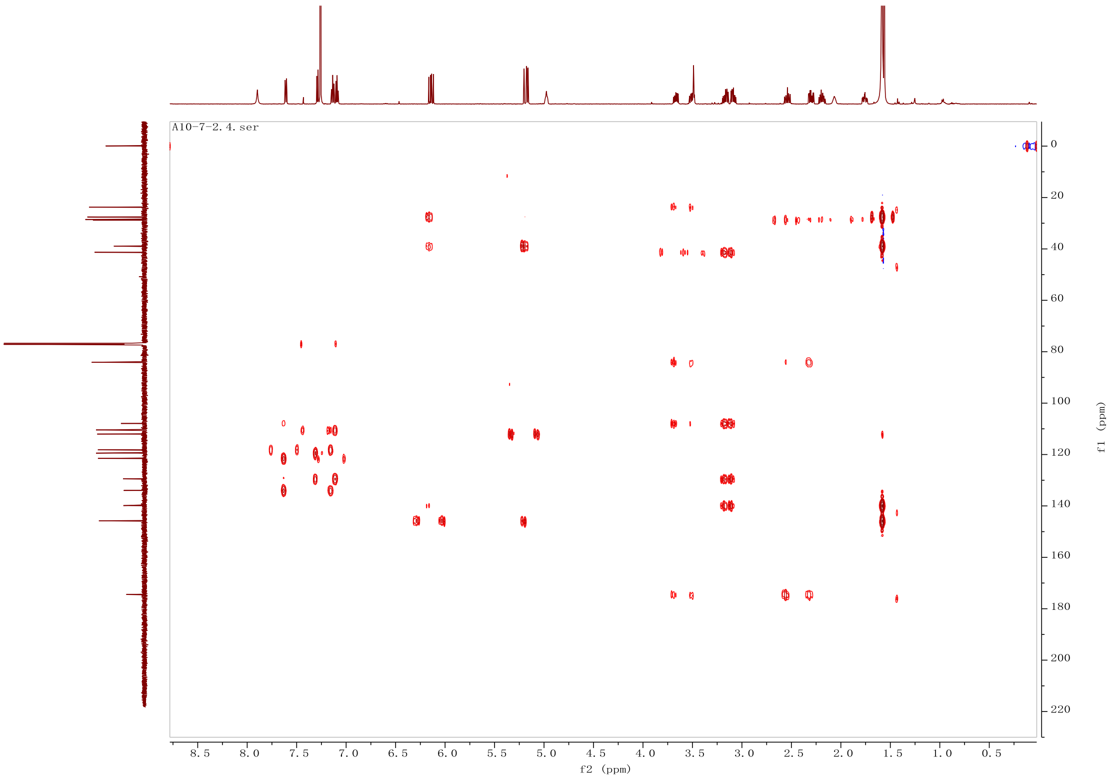

Figure S14. HMBC spectrum (600 MHz, chloroform-d) of **2**

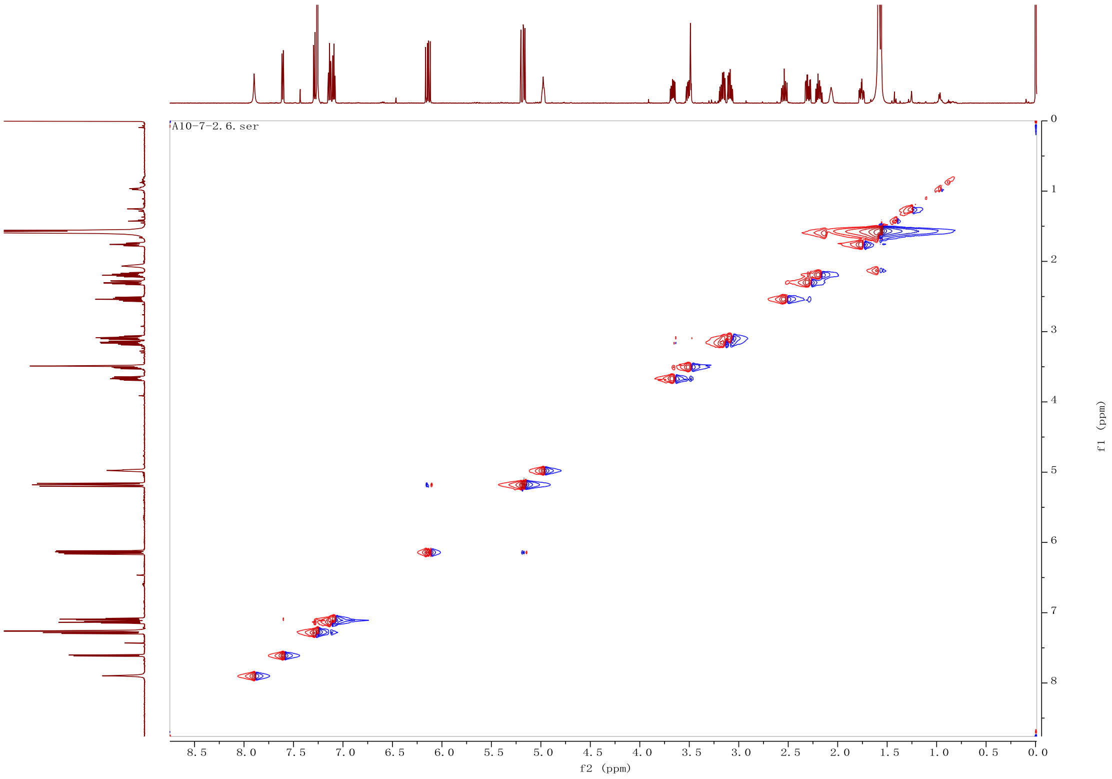

Figure S15. NOESY spectrum (600 MHz, chloroform-d) of **2**

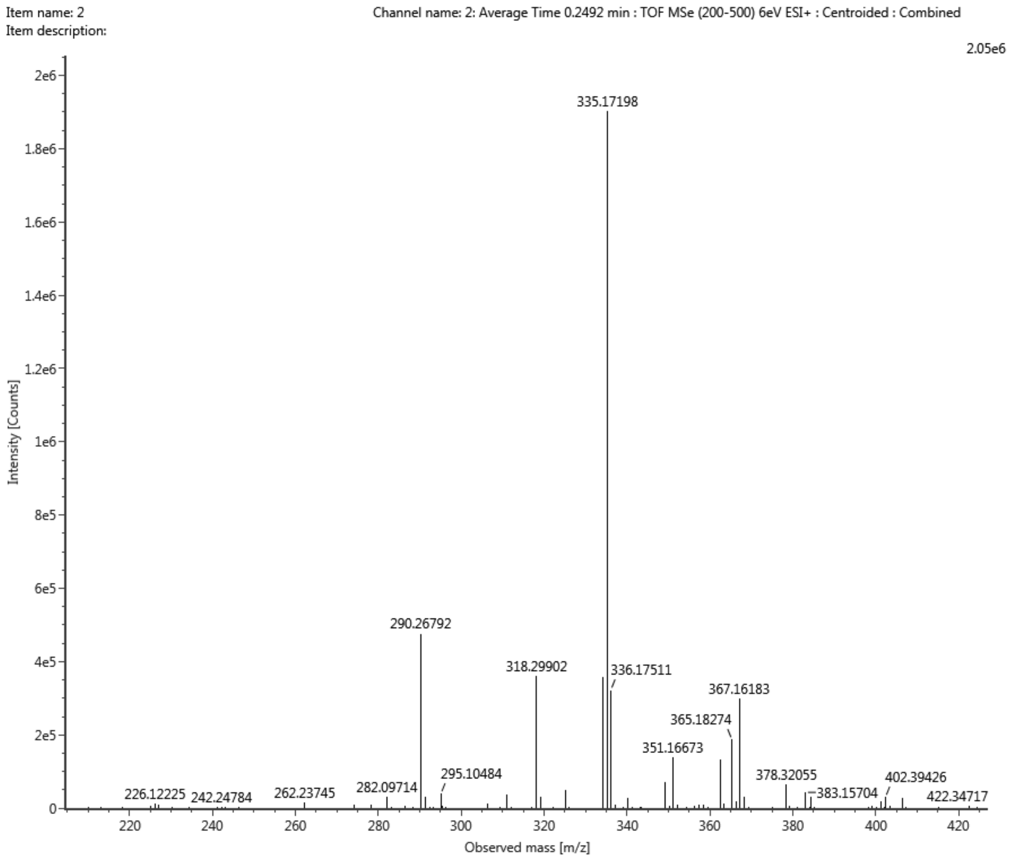

Figure S16. HRESIMS spectrum of **2**

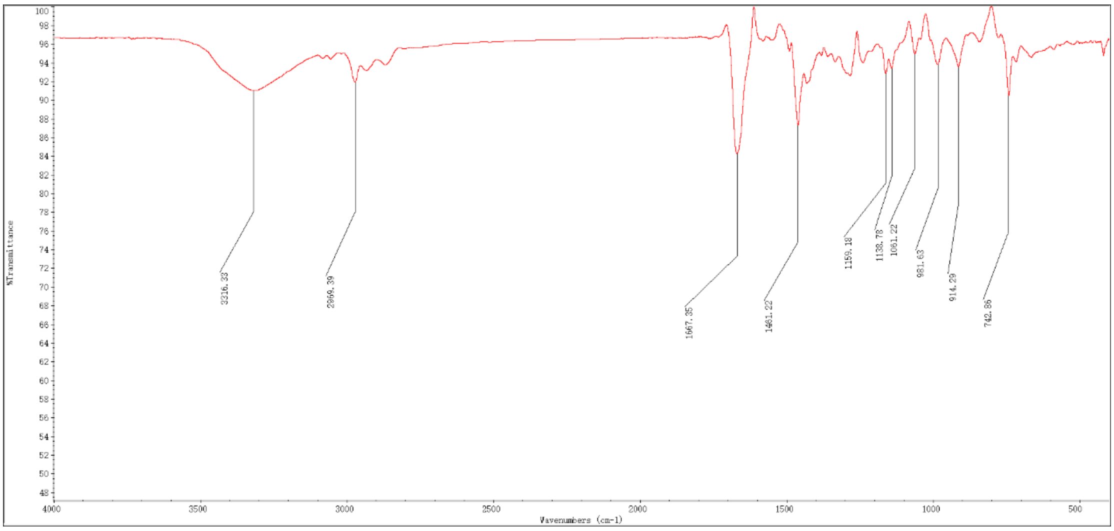

Figure S17. FT-IR spectrum of compound **2**

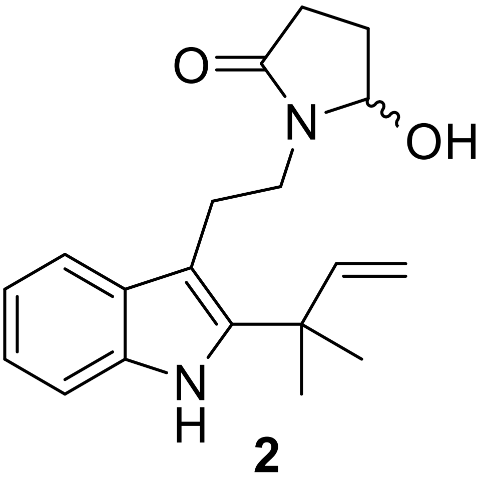

2

Figure S18. Chemcial structure of compound **2**

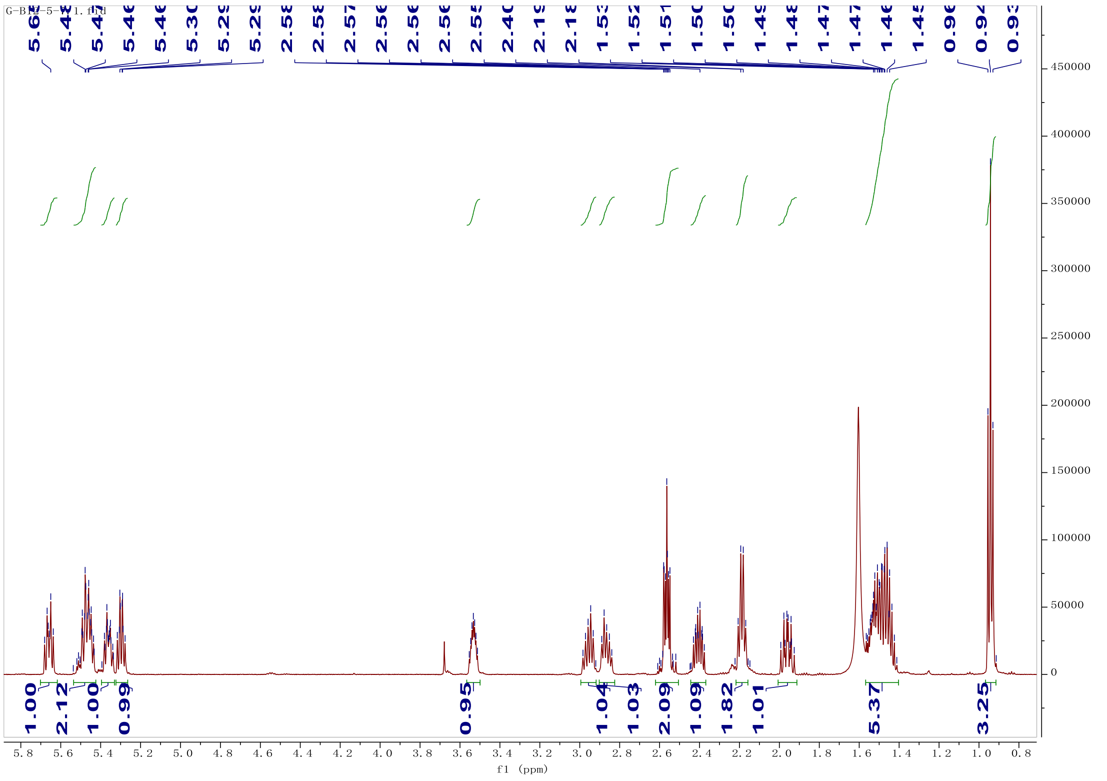

Figure S19. ^{1}H NMR spectrum (600 MHz, chloroform-d) of **3**

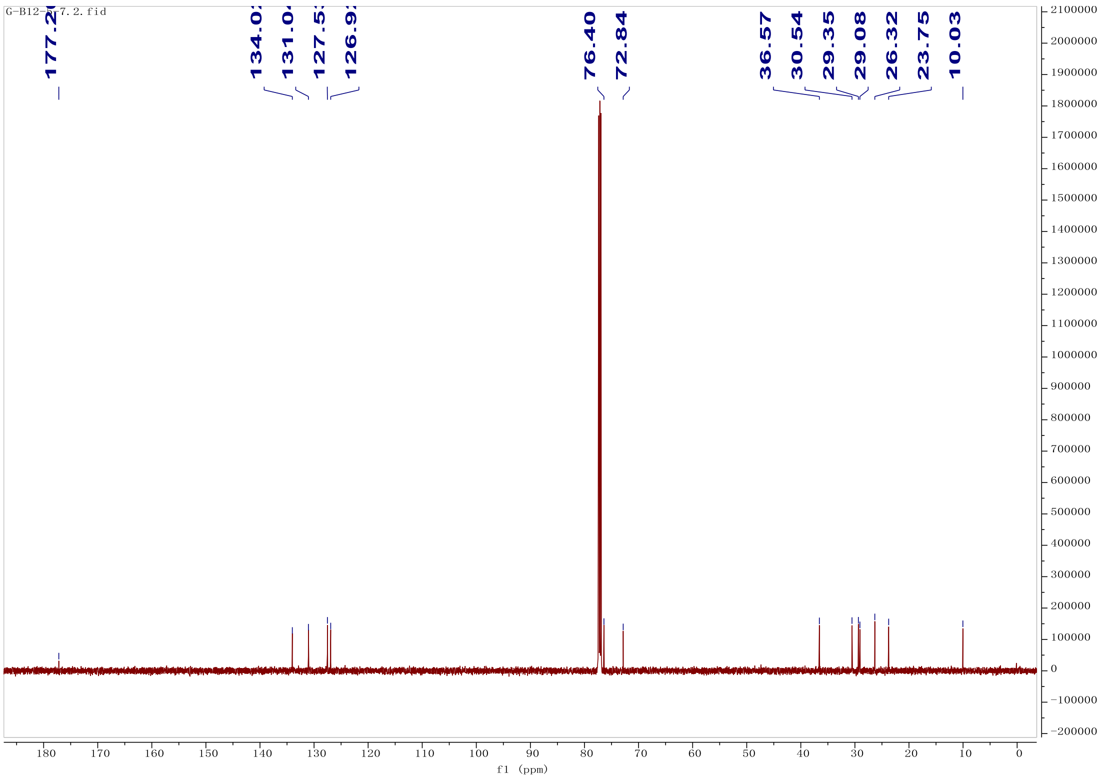

Figure S20. ^{13}C NMR spectrum (600 MHz, chloroform-d) of **3**

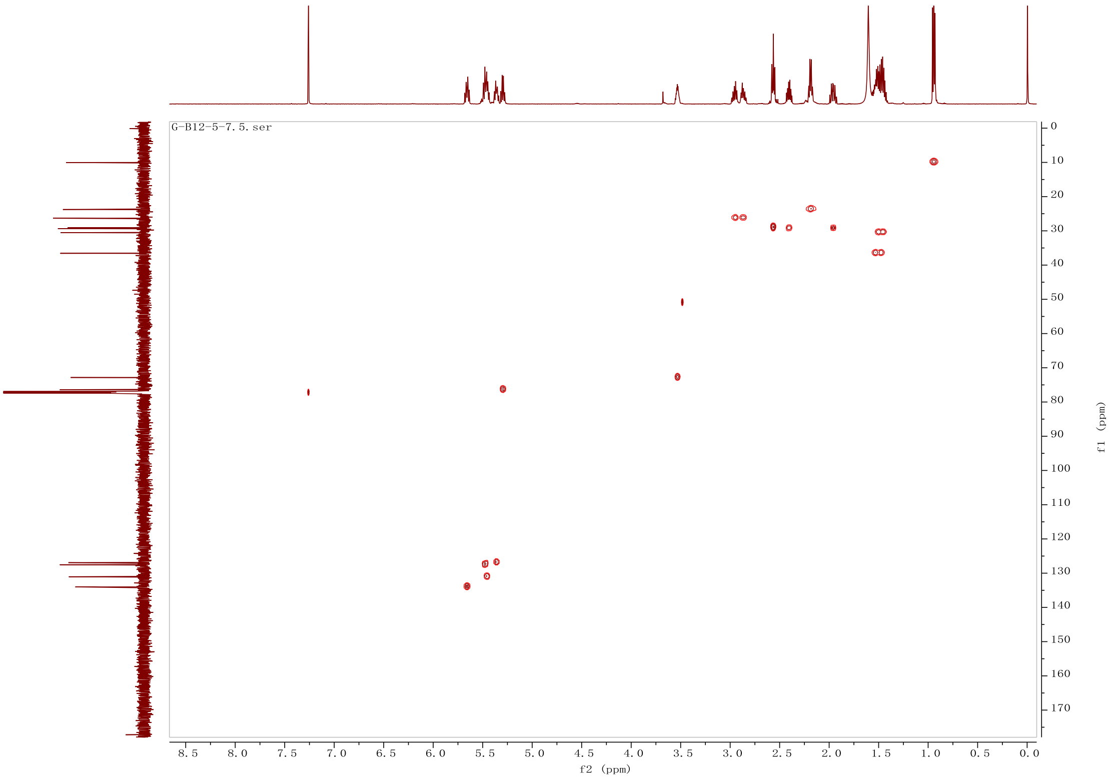

Figure S21. HSQC spectrum (600 MHz, chloroform-d) of **3**

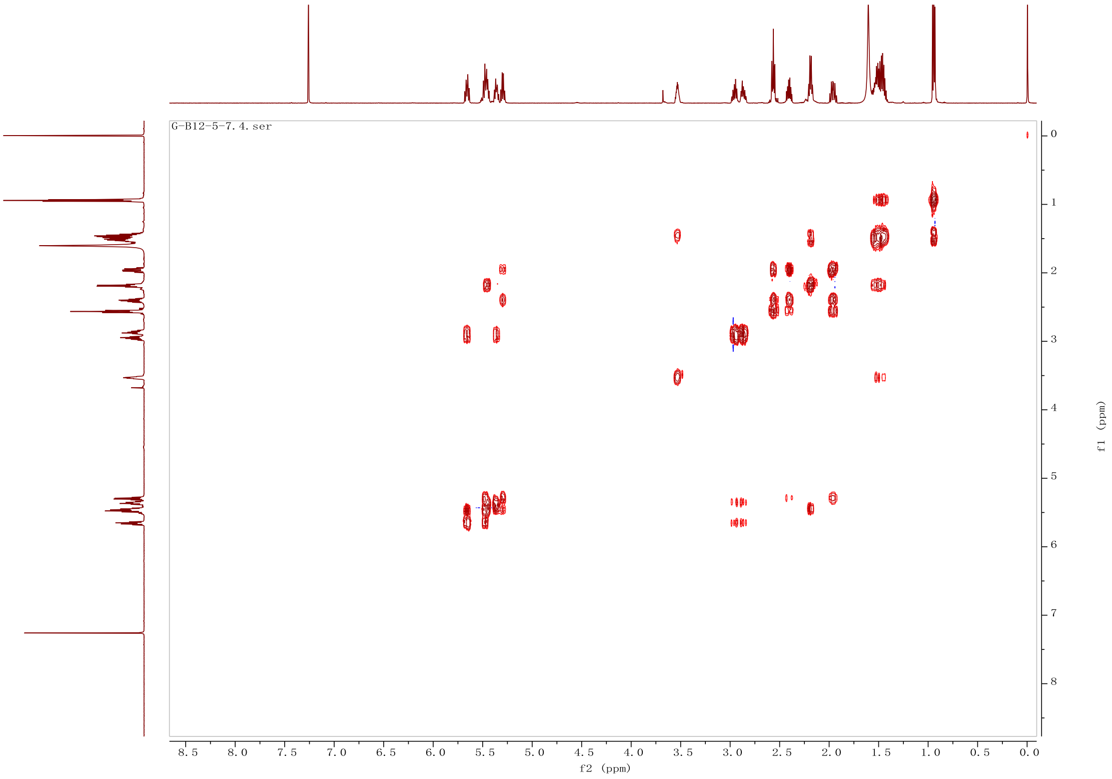

Figure S22. ^{1}H-^{1}H COSY spectrum (600 MHz, chloroform-d) of **3**

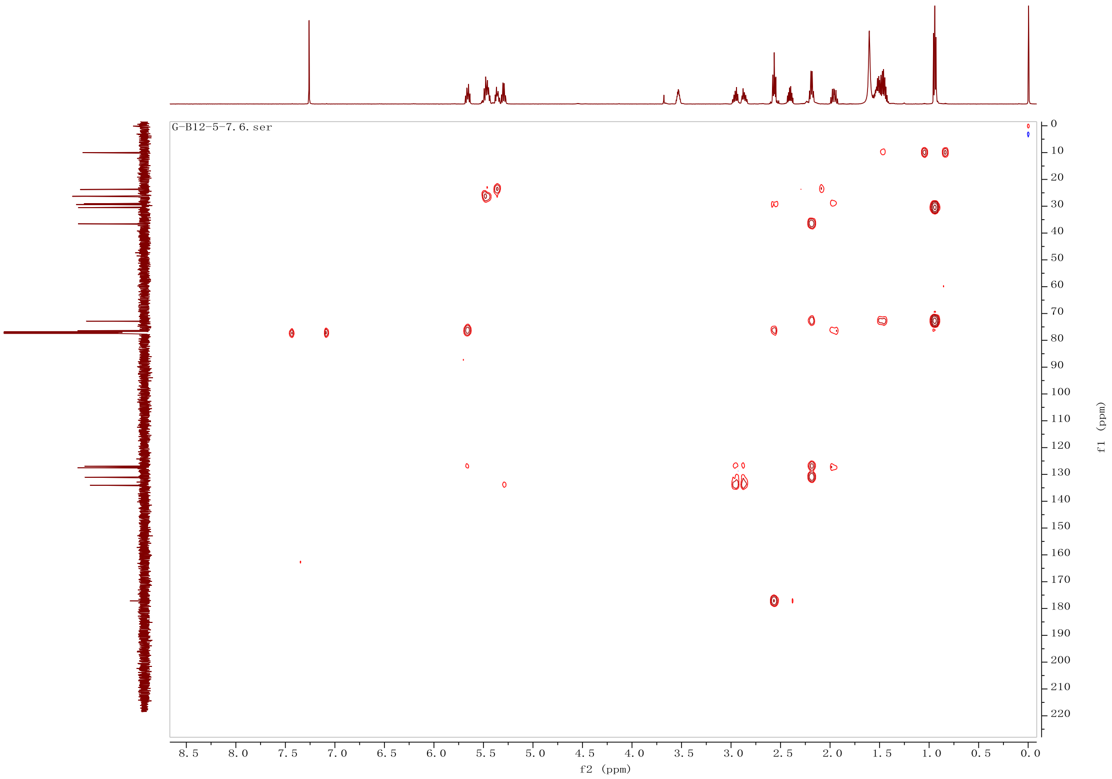

Figure S23. HMBC spectrum (600 MHz, chloroform-d) of **3**

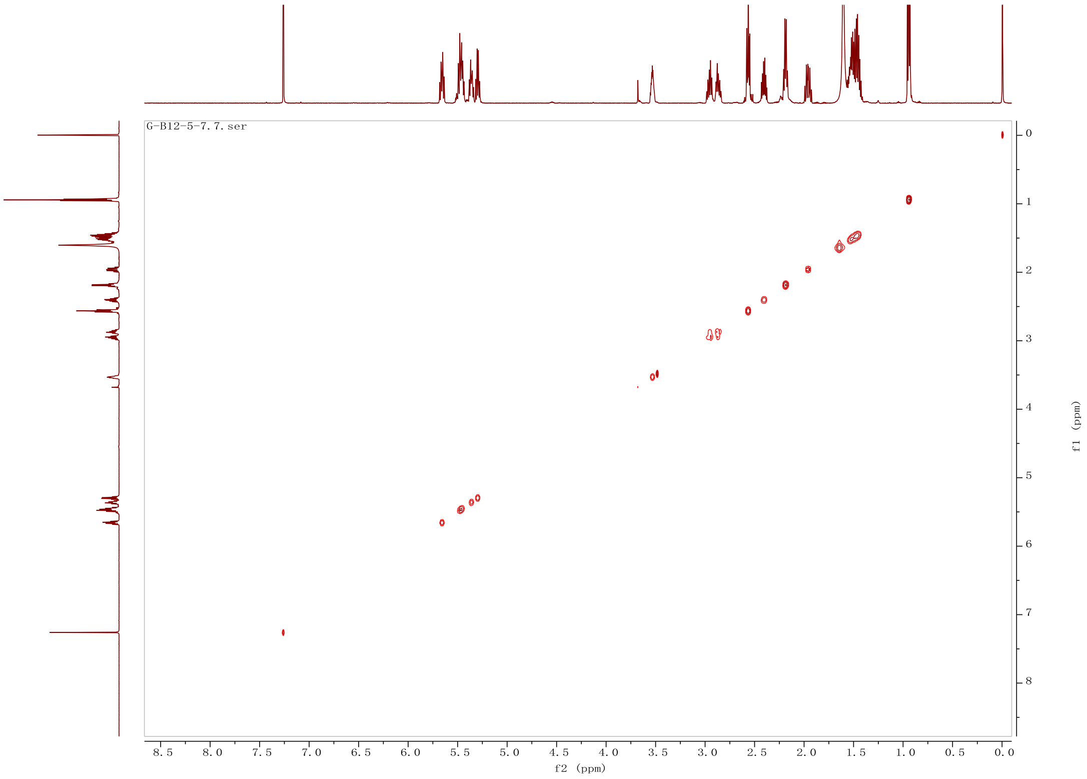

Figure S24. NOESY spectrum (600 MHz, chloroform-d) of **3**

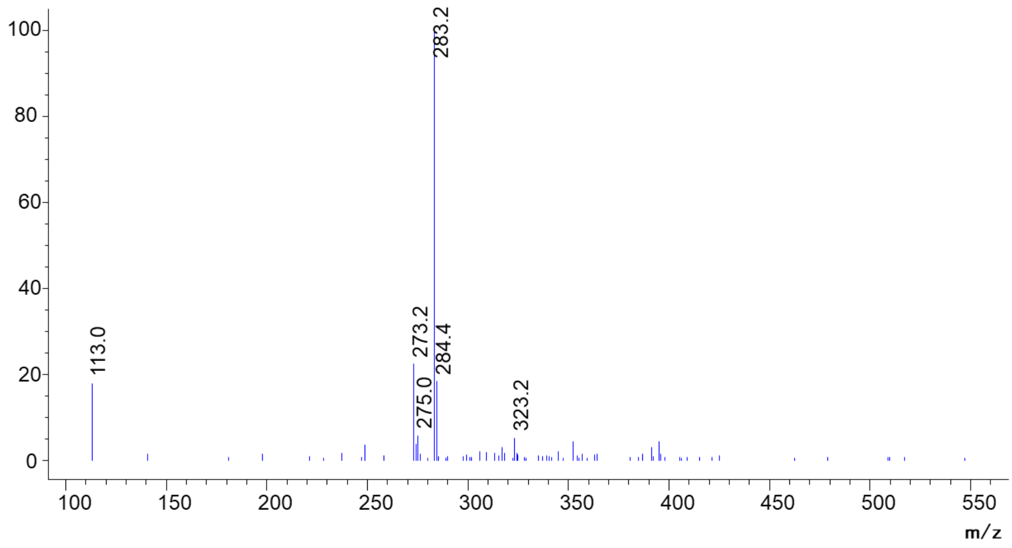

Figure S25. LC-MS spectrum of **3**

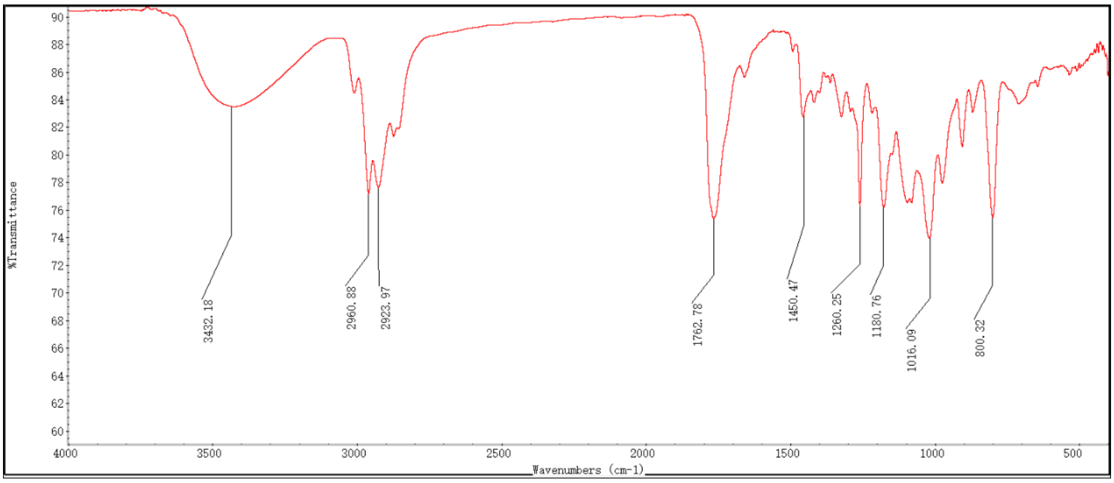

Figure S26. FT-IR spectrum of compound **3**

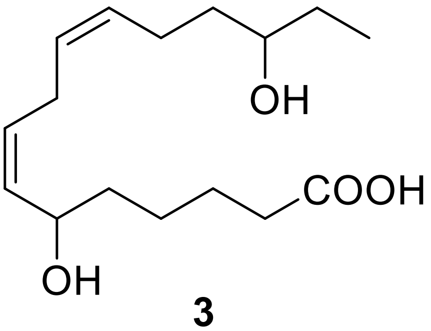

3

Figure S27. Chemcial structure of compounds **3**

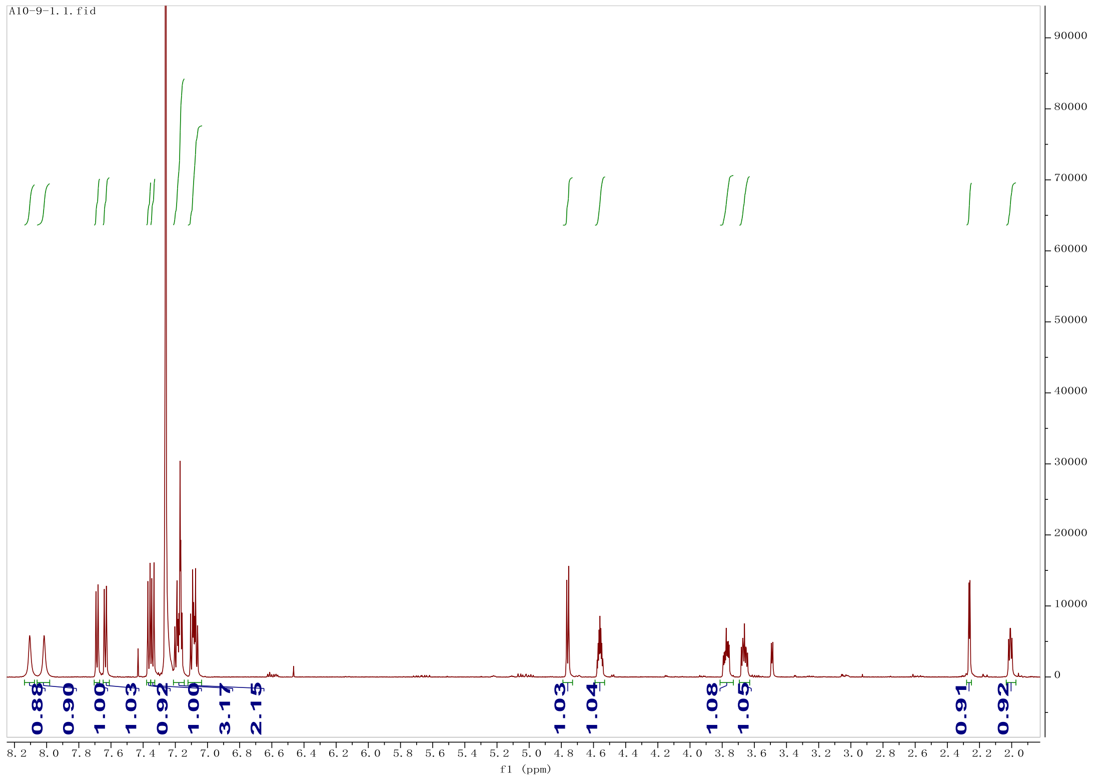

Figure S28. ^{1}H NMR spectrum (600 MHz, chloroform-d) of **4**

^{1}H NMR (600 MHz, chloroform-d) δ 8.11 (s, 1H), 8.02 (s, 1H), 7.69 (dd, J = 8.0, 1.0 Hz, 1H), 7.63 (dd, J = 8.0, 1.0 Hz,1H), 7.36 (d, J = 8.2 Hz, 1H), 7.34 (d, J = 8.1 Hz, 1H), 7.22 – 7.15 (m, 3H), 7.12 – 7.05 (m, 2H), 4.76 (d, J = 7.2 Hz, 1H), 4.56 (tt, J = 7.1, 3.6 Hz, 1H), 3.78 (ddd, J = 10.9, 7.2, 3.5 Hz, 1H), 3.66 (ddd, J = 11.4, 6.7, 4.9 Hz, 1H), 2.26 (d, J = 3.9 Hz, 1H), 2.01 (dd, J = 7.3, 5.1 Hz, 1H).Compound **4** was determined by comparison of its spectroscopic data with fusarindole B in the literature [1].

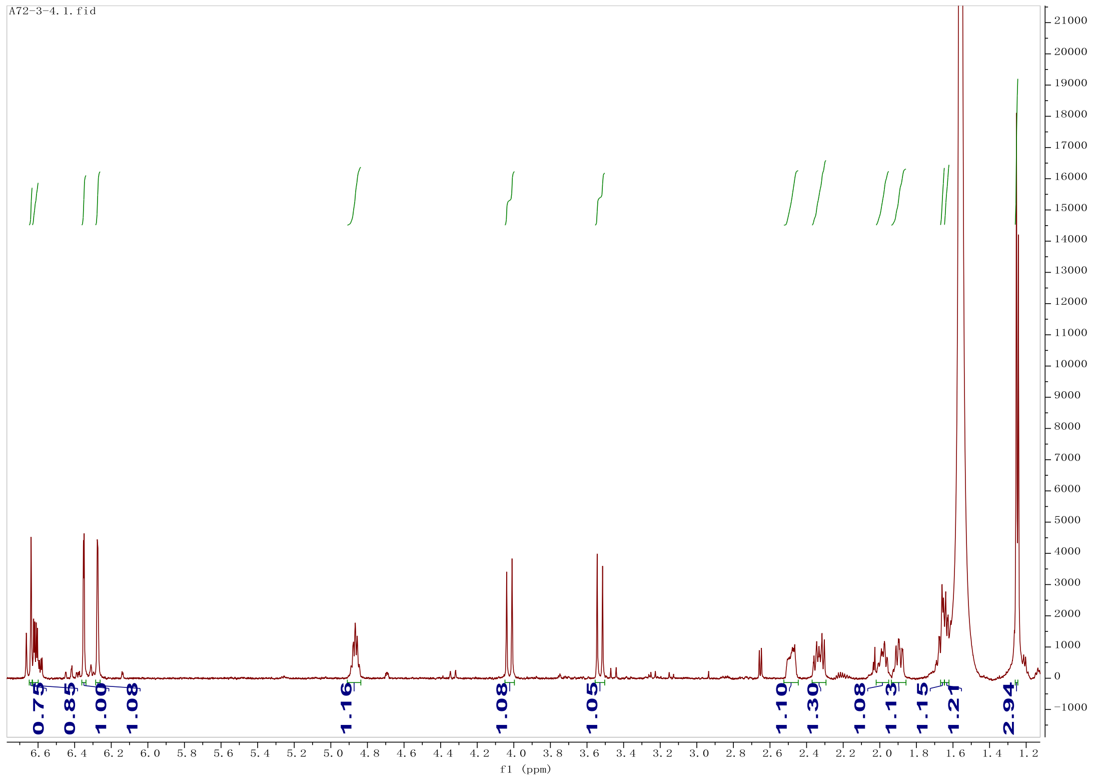

Figure S29. ^{1}H NMR spectrum (600 MHz, chloroform-d) of **5**

^{1}H NMR (600 MHz, chloroform-d) δ 6.64 (d, J = 1.5 Hz, 1H), 6.61 (dd, J = 8.6, 3.8 Hz, 1H), 6.35 (d, J = 2.5 Hz, 1H), 6.27 (d, J = 2.6 Hz, 1H), 4.92 – 4.84 (m, 1H), 4.02 (d, J = 17.7 Hz, 1H), 3.53 (d, J = 17.7 Hz, 1H), 2.47 (d, J = 8.5 Hz, 1H), 2.38 – 2.29 (m, 1H), 1.98 (q, J = 9.3, 7.6 Hz, 1H), 1.93 – 1.86 (m, 1H), 1.66 (d, J = 4.9 Hz, 1H), 1.65 – 1.63 (m, 1H), 1.25 (s, 3H). Compound **5** was determined by comparison of its spectroscopic data with dehydrocurvularin in the literature [2].

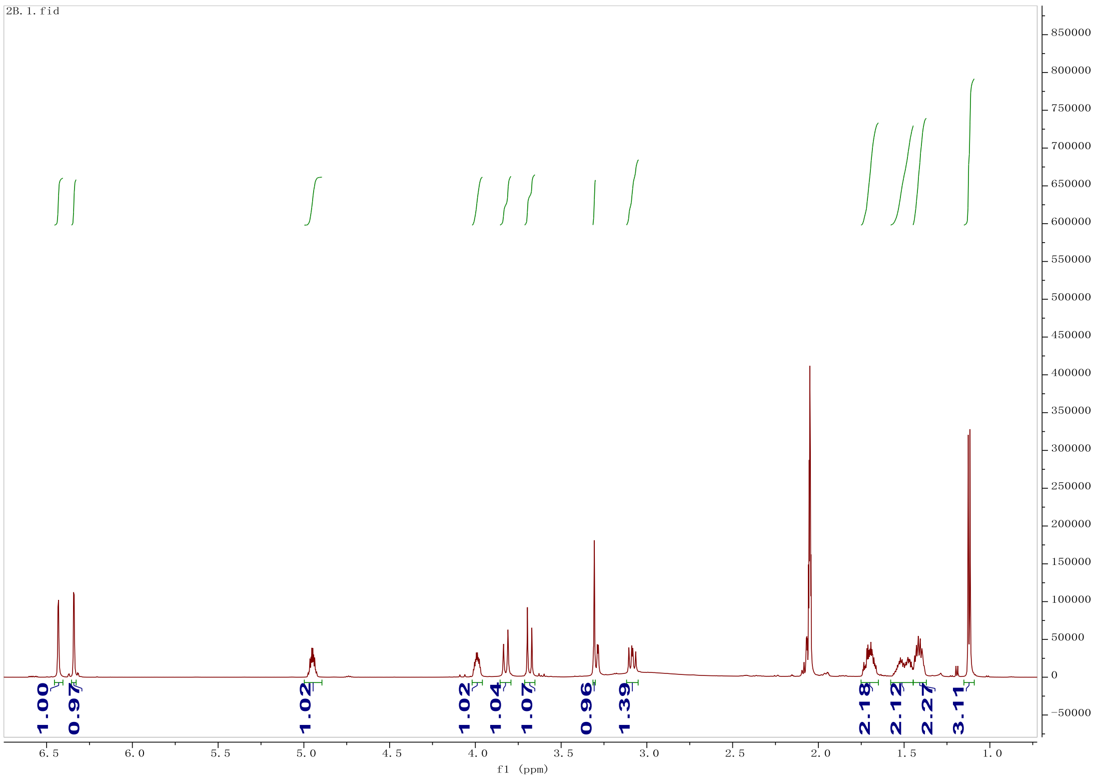

Figure S30. ^{1}H NMR spectrum (600 MHz, acetone-*d*6) of **6**

^{1}H NMR (600 MHz, acetone-*d*6) δ 6.43 (d, J = 2.3 Hz, 1H), 6.34 (d, J = 2.3 Hz, 1H), 4.95 (td, J = 6.6, 3.5 Hz, 1H), 3.99 (ddt, J = 10.4, 7.0, 3.6 Hz, 1H), 3.82 (d, J = 15.4 Hz, 1H), 3.68 (d, J = 15.4 Hz, 1H), 3.31 (d, J = 3.2 Hz, 1H), 3.08 (dd, J = 13.9, 10.0 Hz, 1H), 1.81 – 1.33 (m, 6H), 1.12 (d, J = 6.4 Hz, 3H). Compound **6** was determined by comparison of its spectroscopic data with hydroxycurvularin in the literature [3].

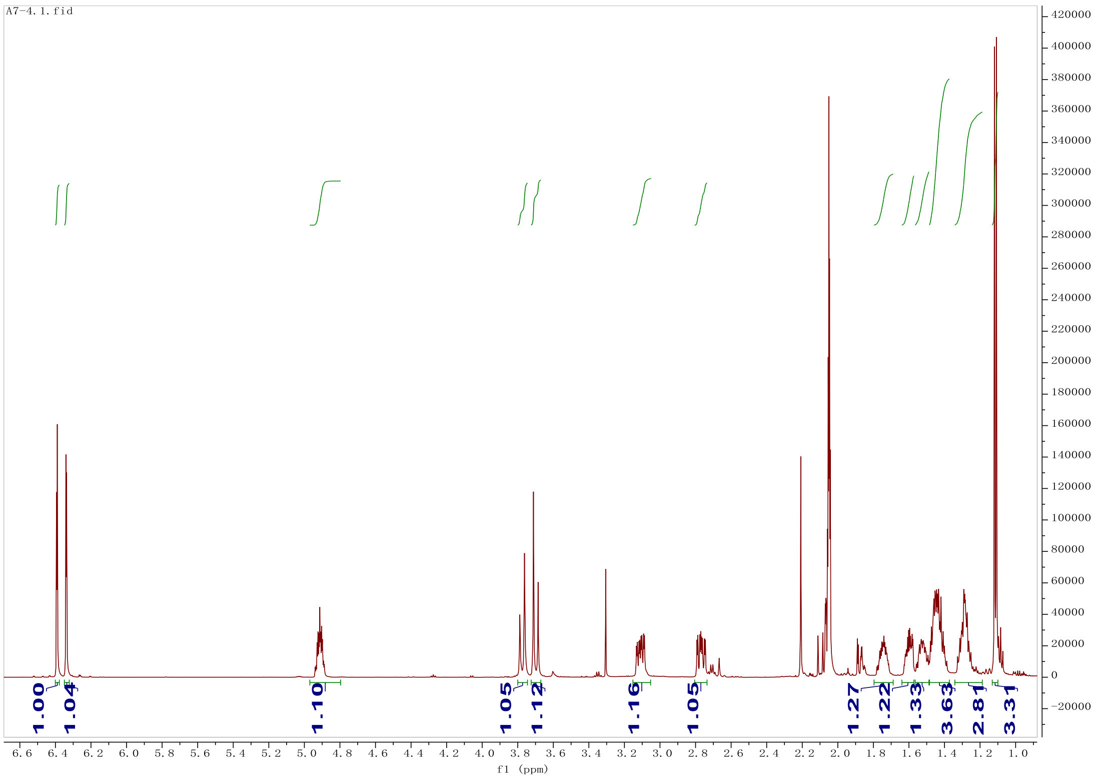

Figure S31. ^{1}H NMR spectrum (600 MHz, acetone-d_6) of **7**

^{1}H NMR (600 MHz, acetone-d_6) δ 6.39 (d, J = 2.3 Hz, 1H), 6.34 (d, J = 2.3 Hz, 1H), 4.91 (dqd, J = 9.0, 6.3, 2.8 Hz, 1H), 3.77 (d, J = 15.7 Hz, 1H), 3.70 (d, J = 15.6 Hz, 1H), 3.11 (ddd, J = 15.5, 8.6, 3.0 Hz, 1H), 2.77 (ddd, J = 15.5, 9.8, 3.0 Hz, 1H), 1.80 – 1.68 (m, 1H), 1.60 (ddt, J = 14.3, 7.9, 3.2 Hz, 1H), 1.56 – 1.49 (m, 1H), 1.49 – 1.37 (m, 3H), 1.35 – 1.21 (m, 2H), 1.11 (d, J = 6.3 Hz, 3H). Compound **7** was determined by comparison of its spectroscopic data with curvularin in the literature [4].

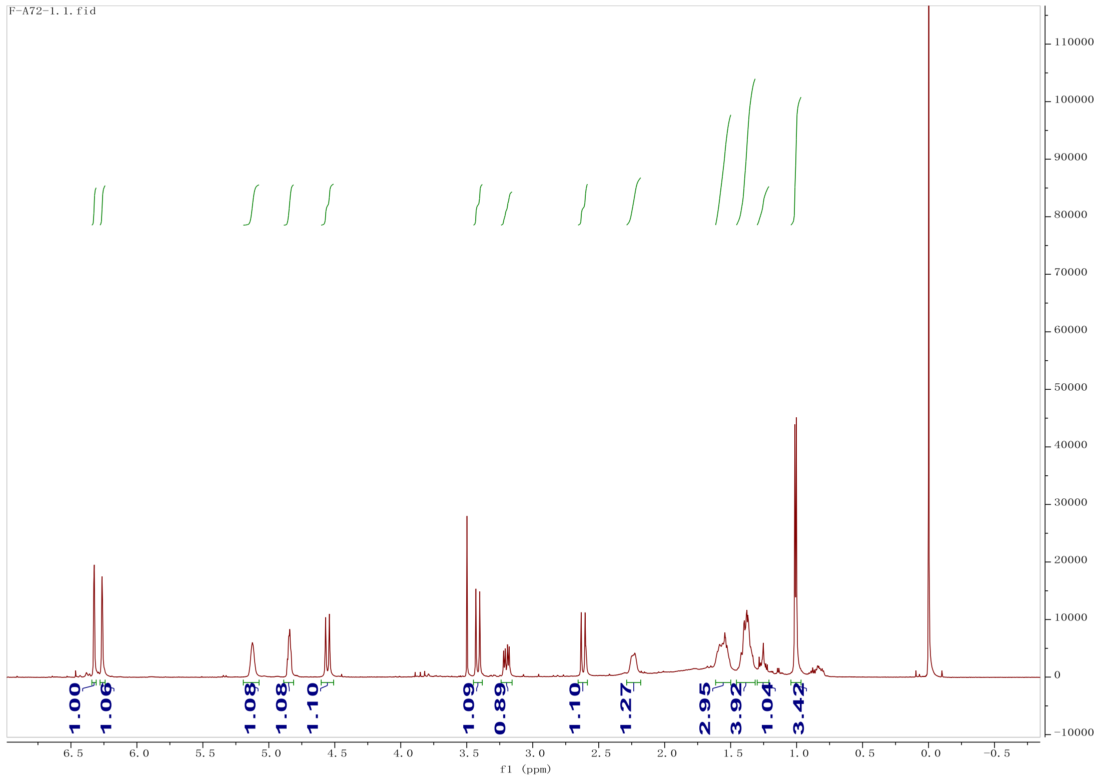

Figure S32. ^{1}H NMR spectrum (600 MHz, chloroform-d) of **8**

^{1}H NMR (600 MHz, chloroform-d) δ 6.32 (d, J = 2.3 Hz, 1H), 6.28 – 6.24 (m, 1H), 5.12 (s, 1H), 4.85 (q, J = 6.1, 5.6 Hz, 1H), 4.56 (d, J = 17.4 Hz, 1H), 3.42 (d, J = 17.4 Hz, 1H), 3.20 (dd, J = 17.7, 7.6 Hz, 1H), 2.62 (d, J = 17.7 Hz, 1H), 2.23 (s, 1H), 1.66 – 1.49 (m, 2H), 1.44 – 1.31 (m, 3H), 1.30 – 1.22 (m, 1H), 1.01 (d, J = 6.6 Hz, 3H). Compound **8** was determined by comparison of its spectroscopic data with curvulopyran in the literature [5].

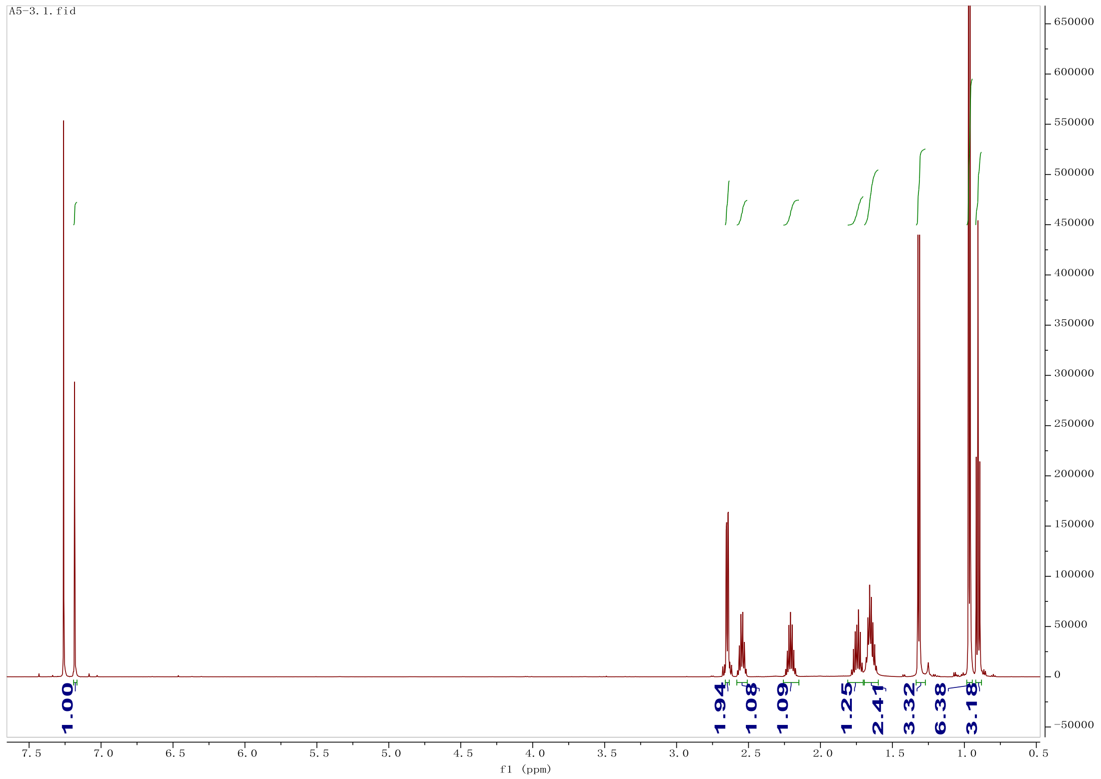

Figure S33. ^{1}H NMR spectrum (600 MHz, chloroform-d) of **9**

^{1}H NMR (600 MHz, chloroform-d) δ 7.18 (s, 1H), 2.65 (dd, J = 7.1, 1.9 Hz, 2H), 2.55 (h, J = 7.1 Hz, 1H), 2.21 (dh, J = 13.5, 6.7 Hz, 1H), 1.75 (dt, J = 13.7, 7.5 Hz, 1H), 1.65 (dp, J = 14.4, 7.3 Hz, 2H), 1.32 (d, J = 7.0 Hz, 3H), 0.97 (d, J = 6.7 Hz, 6H), 0.91 (t, J = 7.4 Hz, 3H). Compound **9** was determined by comparison of its spectroscopic data with (S)-6-(sec-butyl)-3-isobutylpyrazin-2(1H)-one in the literature [6].

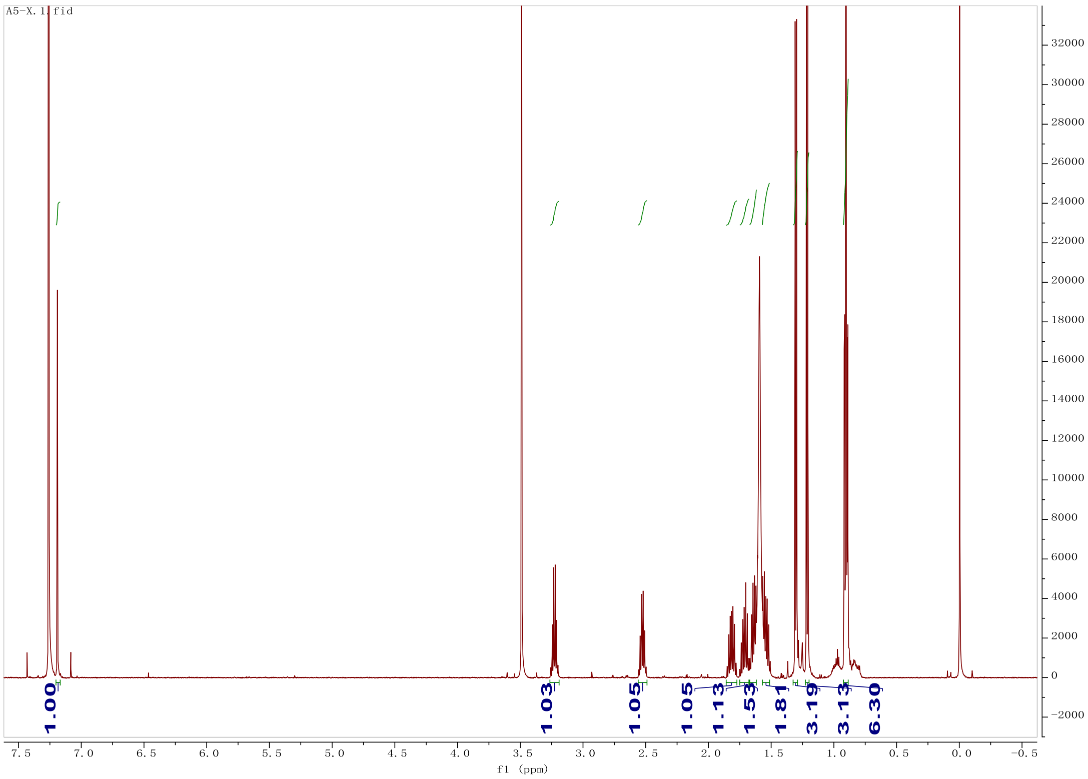

Figure S34. ^{1}H NMR spectrum (600 MHz, chloroform-d) of **10**

^{1}H NMR (600 MHz, chloroform-d) δ 7.19 (s, 1H), 3.23 (h, J = 6.9 Hz, 1H), 2.53 (h, J = 7.1 Hz, 1H), 1.87 – 1.77 (m, 1H), 1.71 (dt, J = 13.8, 7.5 Hz, 1H), 1.64 (dt, J = 14.1, 7.2 Hz, 1H), 1.54 (dt, J = 13.4, 7.3 Hz, 1H), 1.30 (d, J = 7.0 Hz, 3H), 1.21 (d, J = 6.9 Hz, 3H), 0.90 (td, J = 7.4, 2.9 Hz, 6H). Compound **10** was determined by comparison of its spectroscopic data with 3,6-di-sec-butyl-2(1H)-pyrazinone in the literature [6].

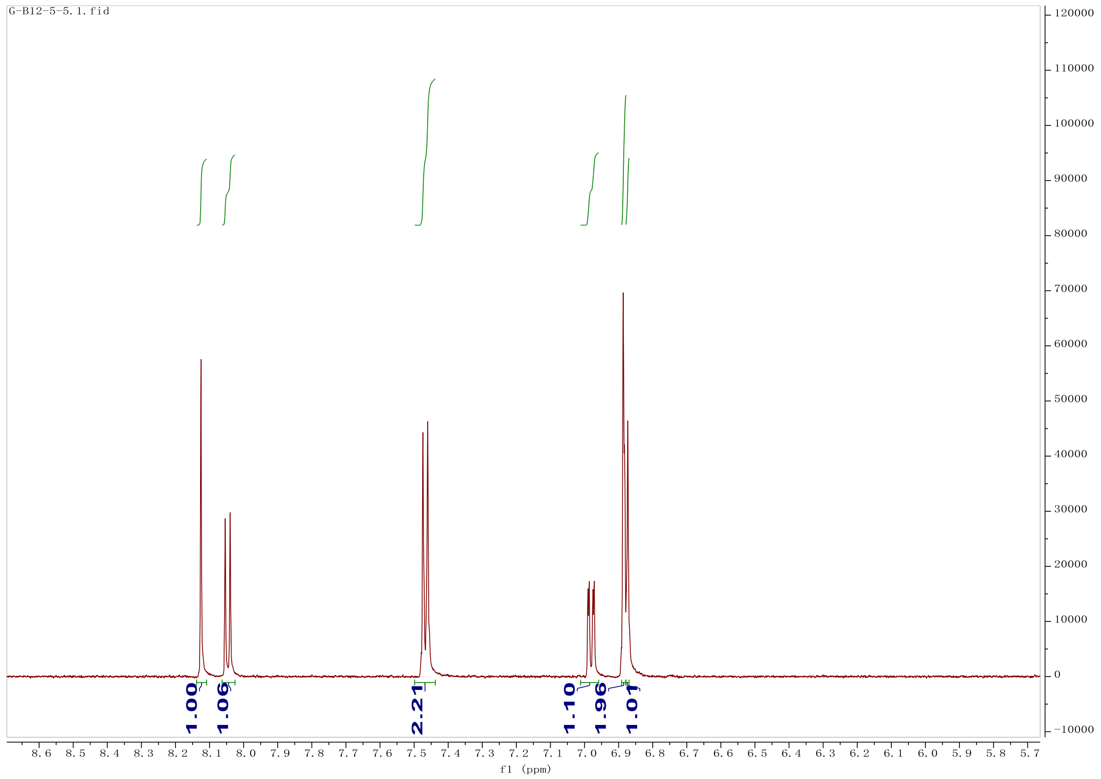

Figure S35. ^{1}H NMR spectrum (600 MHz, acetone-d_6) of **11**

^{1}H NMR (600 MHz, acetone-d_6) δ 8.13 (s, 1H), 8.05 (d, J = 8.7 Hz, 1H), 7.47 (d, J = 8.5 Hz, 2H), 6.98 (dd, J = 8.8, 2.3 Hz, 1H), 6.88 (d, J = 2.6 Hz, 2H), 6.87 (d, J = 2.3 Hz, 1H). Compound **11** was determined by comparison of its spectroscopic data with daidzein in the literature [7].

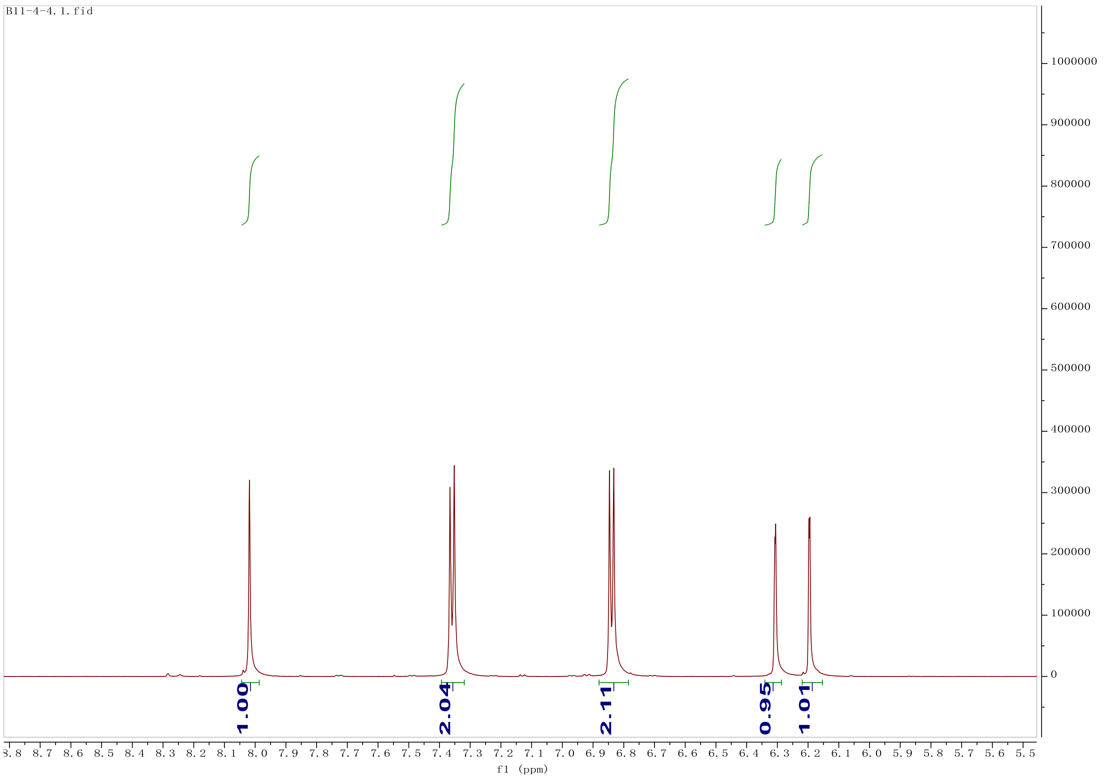

Figure S36.1H NMR spectrum (600 MHz, methanol-d_4) of **12**

^{1}H NMR (600 MHz, methanol-d_4) δ 8.02 (s, 1H), 7.36 (d, J = 8.3 Hz, 2H), 6.84 (d, J = 8.3 Hz, 2H), 6.31 (d, J = 2.1 Hz, 1H), 6.20 (d, J = 2.1 Hz, 1H). Compound **12** was determined by comparison of its spectroscopic data with genistein in the literature [8].

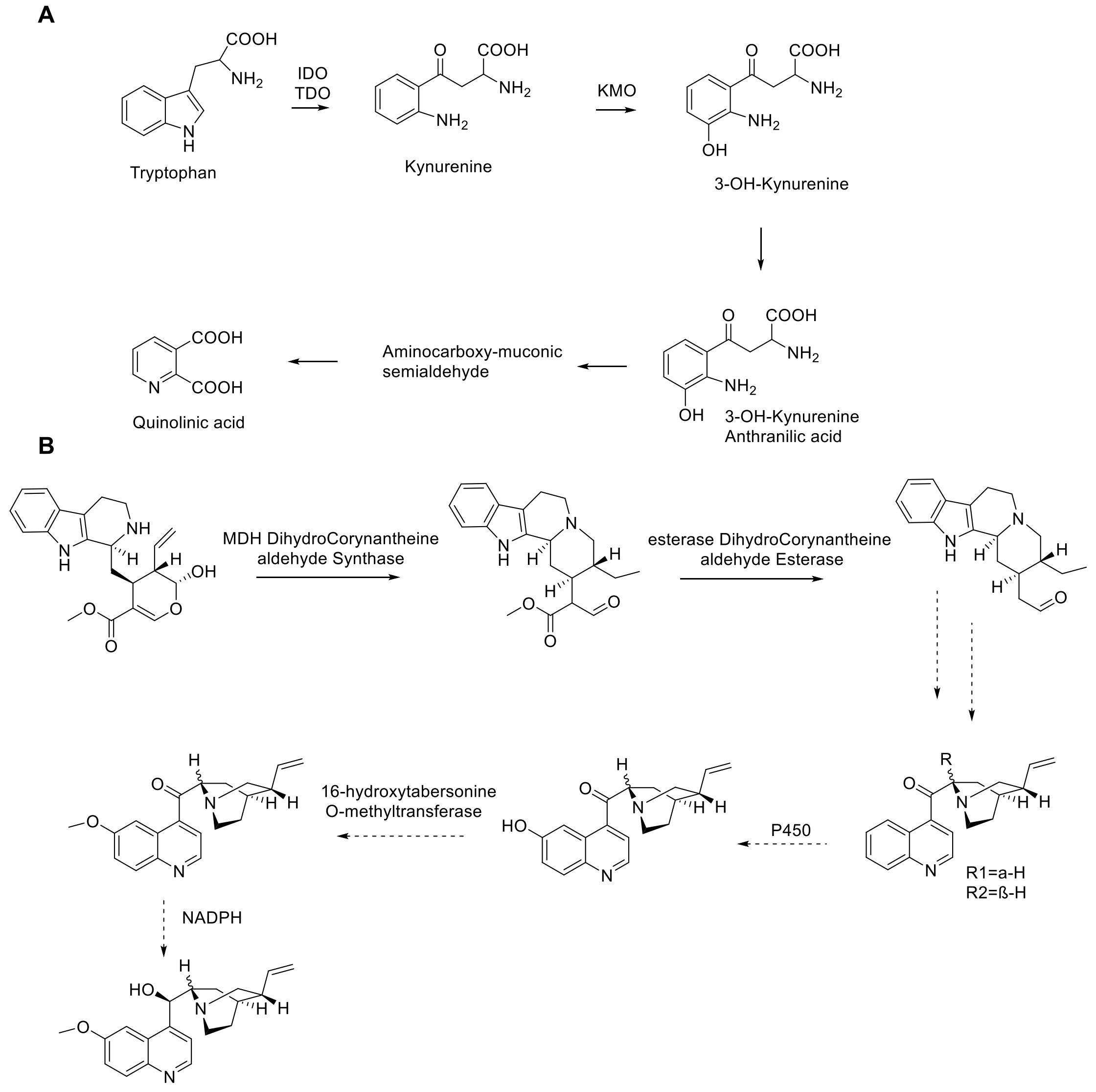

Figure S37. The biosynthetic pathways of the quinoline ring formation

A: The formation of primary metabolic pathways of quinoline rings [9] **B**: The formation of secondary metabolic pathways reported on quinoline rings[10]

Supplementary References

1. Dai XM; Pan HL; Lan WJ; Chen LP; Feng GK; Deng R; Zhu XF; Li HJ. *Phytochemistry.* **2022**, *204*, 113456. doi: 10.1016/j.phytochem.2022.113456

2. Arai, K; Rawlings, B.J; Yoshizawa, Y; Vederas, J.C. *J. Am. Chem. Soc*. **1989**, *111*, 3391-3399. doi: 10.1021/JA00191A042

3. Liu Y; Li Z; Vederas J.C. *Tetrahedron.* **1998**, *54*, 15937-15958. doi: 10.1016/S0040-4020(98)01003-5

4. Ghisalberti E.L; Hockless D.C; Rowland C.Y; White A.H. *Aust. J. Chem.* **1993**, *46*, 571-575. doi: 10.1002/CHIN.199330293

5. Choochuay J; Xu X; Rukachaisirikul V; Guedduaythong P; Phongpaichit S; Sakayaroj J; Chen J; Shen X. *Phytochem. Lett*. **2017**, *22*, 122-127. doi: 10.1016/J.PHYTOL.2017.09.011

6. Shaala L.A; Youssef D.T; Badr J.M; Harakeh S.M. *Molecules.* **2016**, *21*, 1116. doi: 10.3390/molecules21091116.

7. Lee S. J; Baek H. J; Lee C. H; Kim H. P. *Arch. Pharm. Res*. **1994**, *17*, 31-35. doi: 10.1007/BF02978244.

8. Selepe M.A; Drewes S.E; van Heerden F.R. *J. Nat. Prod*. **2010**, *73*, 1680–1685. doi: 10.1021/np100407n. doi:10.1126/science.aaf9794

9. Červenka I; Agudelo L.Z; Ruas J.L. Science. **2017**, *357*, 6349. doi:10.1126/science.aaf9794

10. Trenti F； Yamamoto K； Hong B； Paetz C； Nakamura Y； O'Connor S.E. Early and Late Steps of Quinine Biosynthesis. Org. Lett. **2021**, *23*, 1793-1797. doi:10.1021/acs.orglett.1c00206
